# A Neutral Three‐Membered 2π Aromatic Disilaborirane and the Unique Conversion into a Four‐Membered BSi_2_N‐Ring

**DOI:** 10.1002/anie.202009638

**Published:** 2020-10-12

**Authors:** Samir Kumar Sarkar, Rinkumoni Chaliha, Mujahuddin M. Siddiqui, Samya Banerjee, Annika Münch, Regine Herbst‐Irmer, Dietmar Stalke, Eluvathingal D. Jemmis, Herbert W. Roesky

**Affiliations:** ^1^ Institut für Anorganische Chemie Universität Göttingen Tammannstrasse 4 37077 Göttingen Germany; ^2^ Inorganic and Physical Chemistry Department Indian Institute of Science Bangalore 560012 India; ^3^ Solid State and Structural Chemistry Unit Indian Institute of Science Bangalore 560012 India

**Keywords:** amidinato ligands, aromaticity, disilaborirane, molecular orbitals

## Abstract

We report the design, synthesis, structure, bonding, and reaction of a neutral 2π aromatic three‐membered disilaborirane. The disilaborirane is synthesized by a facile one‐pot reductive dehalogenation of amidinato‐silylene chloride and dibromoarylborane with potassium graphite. Despite the tetravalent arrangement of atoms around silicon, the three‐membered silicon‐boron‐silicon ring is aromatic, as evidenced by NMR spectroscopy, nucleus independent chemical shift calculations, first‐principles electronic structure studies using density functional theory (DFT) and natural bond orbital (NBO) based bonding analysis. Trimethylsilylnitrene, generated in situ, inserts in the Si−Si bond of disilaborirane to obtain a four‐membered heterocycle 1‐aza‐2,3‐disila‐4‐boretidine derivative. Both the heterocycles are fully characterized by X‐ray crystallography.

## Graphical abstract

A neutral 2π electronic three‐membered aromatic disilaborirane is reported where aromatic stabilization arises from the overlap of a p orbital on boron and two σ* MOs of silicon. Also, the intriguing reactivity of the disilaborirane is studied and a four‐membered heterocycle is obtained.

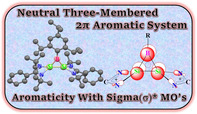

The synthesis of three‐membered aromatic rings has been a fascinating topic for the chemical community during the last decades. Aromaticity and Hückel 4n+2 rule continue its diversity with the smallest, largest, homo‐, hetero‐, Möbius, all‐carbon, organometallic‐ and so on, but most of these involve overlap of p, d and f orbitals.[^1^, ^2^, ^3^] A challenging question is it possible to generate a carbon‐free neutral three‐membered aromatic ring. The neutral three‐membered aromatic compound with 2π electrons is one of the longest quests to inorganic, organic and theoretical chemists.[^1^, ^4^] Most of the reported small aromatic species are anionic or cationic.[^4^, ^5^, ^6^, ^7^, ^8^, ^9^, ^10^, ^11^] Recent reports of the carbon‐free aromatic systems all involve charged species: B_3_^−^ (observed in gas phase),^[6]^ Li_3_^+^( observed in gas phase),^[7]^ [B_3_(CO)_3_]^+^ (observed in gas phase),^[8]^ [B_3_(NCy_2_)_3_]^2−^ (characterized by X‐ray),^[9]^ [SiRSi_2_R′_2_ (R=Si^t^Bu_3_, R′=SiMe^t^Bu_2_)]^+^ (characterized by X‐ray),^[10]^ [Ge_3_R_3_, R=Si^t^Bu_3_]^+^ (characterized by X‐ray).^[11]^ To develop carbon‐free small neutral Hückel π systems with orbitals beyond p, d and f, unique design strategies must be assembled. Here, we present a novel approach where two of the three p orbitals are replaced by σ* MOs to generate a neutral 2π aromatic three‐membered disilaborirane.

To synthesize carbon‐free neutral three‐membered 2π aromatic system, we replace one of the CH group of the classical cyclopropenyl cation by isoelectronic BR group and the remaining two CH groups by two amidinato‐silylene groups to retain the 2π aromaticity, leading to the formation of a disilaborirane.^[12]^ We envision that the extra electrons from the two amidinato‐ligands will populate the stable delocalized π MO resulting from the 2p orbital of boron atom and the two σ* MOs on silicon atoms in the neutral Si_2_B three‐membered ring.^13^ The two π electrons will conjugate in the field of the three nuclei of Si‐B‐Si. Accordingly, we design and synthesize the three‐membered disilaborirane (Scheme 1). A 2:1:4 molar ratio of amidinato‐silylene chloride [LSi‐Cl; L=PhC(NtBu)_2_], dibromo(2,4,6‐triisopropylphenyl)borane and KC_8_ is reacted in THF at −78 °C and allowed to warm up slowly to room temperature. The reaction mixture is evaporated and dry toluene is added. The resultant red solution is filtered in an inert atmosphere and reduced to 5 mL under vacuum. Red block‐shaped crystals of **1** are obtained in 67 % yield at −30 °C after three days. The structure of **1** is fully characterized by NMR (^1^H, ^13^C, ^11^B, ^29^Si, Figures S1–4) and mass spectrometry (LIFDI, Figures S5 –6, SI) techniques. The ^1^H and ^13^C NMR spectral pattern and the relative integration values of **1** are in agreement with the molecular structure. The ^11^B NMR spectrum of **1** shows a singlet at *δ*=11.09 ppm, which falls in the aromatic region of ^11^B NMR.^[14]^ The ^29^Si NMR spectral resonance appears at *δ*=−71.03 ppm and shifts to upfield in comparison to amidinato‐silylene chloride (*δ*=14.6 ppm).

**Scheme 1:**
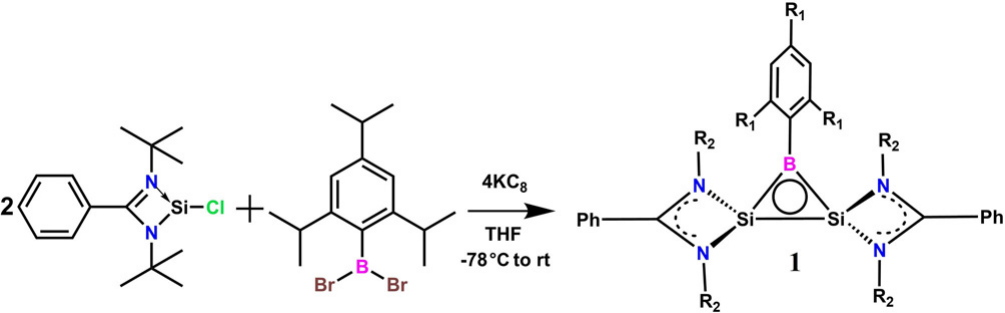
Synthetic scheme for the preparation of **1** (R_1_=isopropyl; R_2_=tert‐butyl).

The structure of **1** is further confirmed by single‐crystal X‐ray diffraction studies (Figure 1). Compound **1** crystallizes in the monoclinic space group *P*2_1_/*c* with one molecule of **1** and one and a half toluene molecule in the asymmetric unit. The heterocycle is an isosceles triangle with identical Si−B bond lengths (1.9190(13), 1.9186(13) Å). Each silicon is coordinated by one bidentate amidinato ligand and the bridging RB (*R*=2,4,6‐triisopropylphenyl) group to form the three‐membered ring. The amidinato ligands are arranged almost perpendicular to the three‐membered ring (86.48(5)° and 83.14(5)°) and the 2,4,6‐triisopropylphenyl substituent on boron is rotated 14.27(5)° out of the heterocycle. The Si1‐Si2 bond length in **1** is 2.1877(5) Å, in the range for a Si‐Si double bond (2.12–2.25 Å) ^[15]^ and comparable to the bond lengths in the (di‐t‐butyl(methyl)silyl)bis(tri‐t‐butylsilyl)cyclotrisilenylium cation (2.211(3)–2.221(3) Å).^[10]^ The average Si−N bond length of 1.89 Å is at the upper limit of the range for Si‐N distances of the amidinato ligand.^[16]^

**Figure 1:**
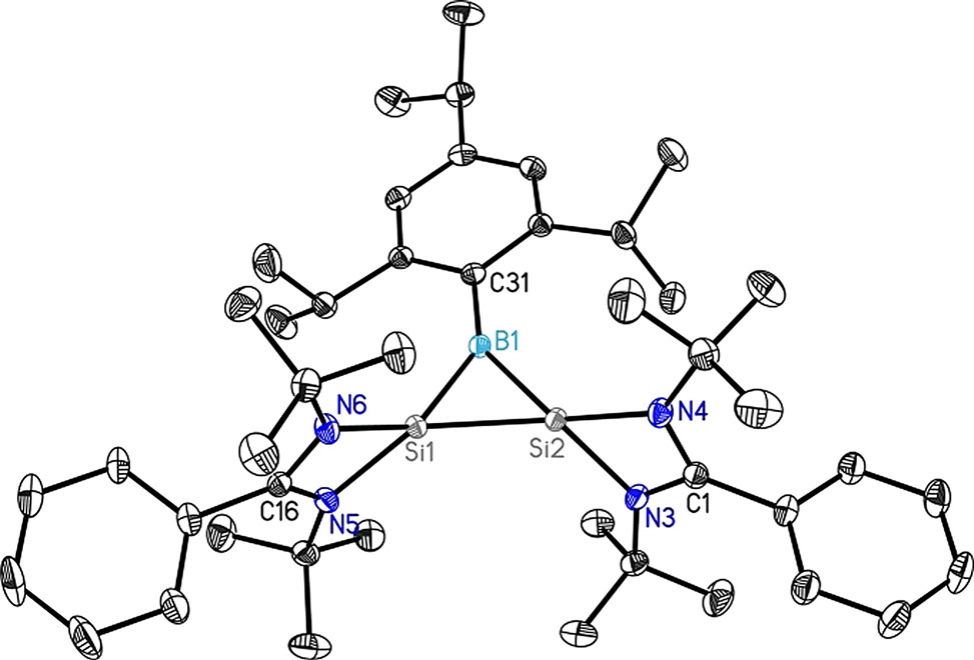
Molecular structure of **1** with anisotropic displacement parameters at the 50 % probability level. Hydrogen atoms are omitted for clarity. Selected bond lengths [Å] and angles of **1** [°]; Si1–Si2 2.1877(5), Si1–B1 1.9190(13), Si2–B1 1.9186(13), Si1–N6 1.8909(10), Si1–N5 1.8921(10), Si2–N3 1.8834(10), Si2–N4 1.8961(10); Si1‐B1‐Si2 69.51(4), B1‐Si2‐Si1 55.25(4), Si2‐Si1‐B1 55.24(4). For structural details and all CCDC numbers see the Supporting Information.

Detailed electronic structure studies of **1** using First‐principles Density Functional Theory with BP86 functional and 6‐31G (d,p) basis set support our qualitative analysis of the electronic structure.^[17]^ The geometric parameters of the optimized structure of **1** are in good agreement with the crystal structure data (Table S4, SI). The HOMO (highest occupied molecular orbital) corresponds to the delocalized π MO, largely localized on the two Si and B (Figure 2 a, Table S5, SI). The contribution of nitrogen to the σ* MOs of π symmetry is antibonding but small. The HOMO‐1 and HOMO‐2 are the Walsh orbitals in the σ‐plane (Figures 2 b and c), just as observed in cyclopropenyl cation.

**Figure 2:**
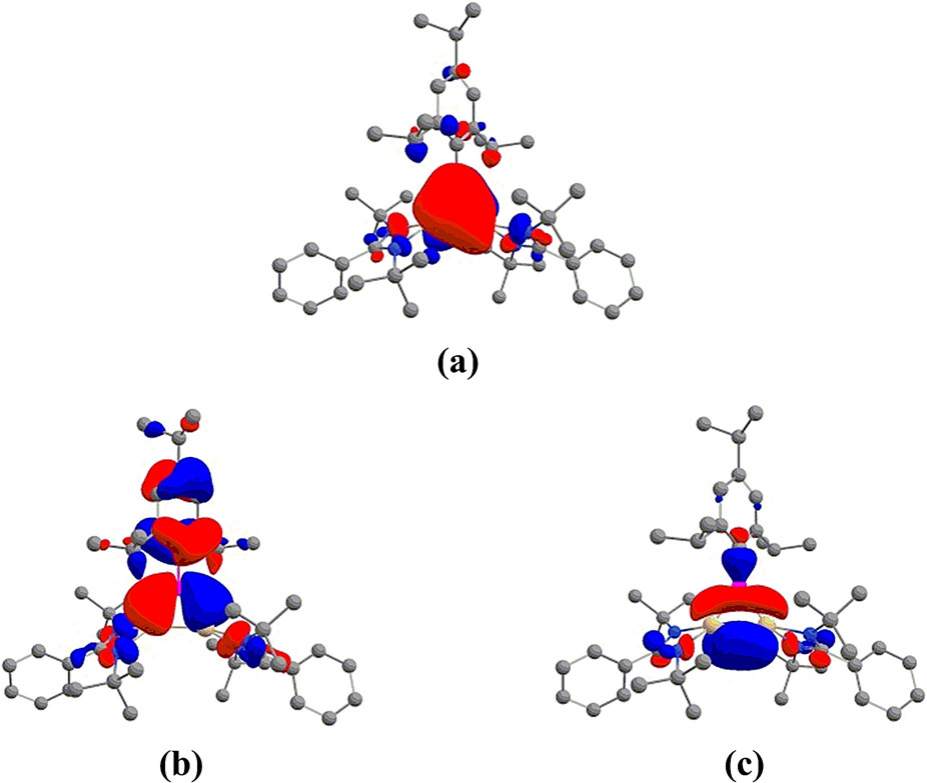
a) The delocalized πMO, HOMO; Walsh orbitals b) HOMO−1 and c) HOMO−2 of **1**. Hydrogen atoms are omitted for clarity.

The multiple bond character of both Si−Si bond and Si−B bonds are reflected in the NBO based Wiberg Bond Index (WBI)^[17]^ values of 1.128 and 1.288 (Table S6, SI). This is to be compared to the WBIs of 1.406 for Si_3_H_3_^+^, 1.903 for H_2_SiSiH_2_ and 1.893 for H_2_SiBH_2_^−1^. We have sought to find the π alone contribution to Si‐Si and Si−B bonding in **1** by NBO‐WBI and the model systems. The total WBI and their π components are given in Table S6 (SI). Thus, the total Si‐Si WBI of 1.128 in **1** is divided into the π component of 0.208 and σ component of 0.920. The corresponding numbers for Si‐B are 0.345 for π and 0.943 for σ. The σ components in Si_3_H_3_^+^, H_2_SiSiH_2_ and H_2_BH_2_^−1^ (0.970, 1.076, 1.036) are comparable. However, the corresponding π components (0.436, 0.827, 0.858) indicate the extent to which the π components in **1** is reduced due to π‐delocalization. Therefore, description of **1** as a 2π aromatic with two σ* MOs of π symmetry and a pure p orbital is justified undoubtedly. The transfer of electrons from the two (NCN)Si fragments to the delocalized πMO (HOMO) is reflected in the total charges obtained from NBO analysis (Table S7, SI). Boron fragment has a charge of −1.005, clearly indicating that the two electrons from the amidinato‐ligands are delocalized in the π MO, HOMO. Correspondingly a reasonably large dipole moment of 6.451 Debye is calculated for **1**.

The extend of π aromaticity can also be gauged by Nucleus Independent Chemical Shift (NICS_zz_(1))^[17]^ values. A NICS_zz_(1) value of −8.1 is computed for Si_2_B ring in **1** (Table S9, SI) compared to the value of −9.1 calculated for the 2π aromatic analogue (SiH)_2_BH and of −8.3 for the closest 2π aromatic with two σ* MOs of π symmetry ((SiH_2_)_2_BH^−2^). Corresponding values for C_3_H_3_^+^ and Si_3_H_3_^+^ are −28.7 and −7.1, respectively.

In order to assess the variation of π delocalization above the ring center of structure **1**, isotropic chemical shifts and its in‐plane and out‐of‐plane components are scanned and plotted, (Figure 3).^[17]^ A minimum in NICS_zz_ at a nonzero r value is indicative of aromatic π delocalization (red curve).^[18]^ These scans are also done for model complexes (SiH_2_)_2_BH^2−^, **(3 b)^2−^** and (CN_2_H_3_Si)_2_BH, (**4)** (Figure S17). In all the three cases, the out‐of‐plane component of NICS (NICS_zz_) curve decreases from 0 Å and reaches a minimum in between 0.8 Å to 1.4 Å. The curve continues to be in the negative region for a considerable distance. This indicates a delocalization of π cloud above the three‐membered rings.^18^ NICS‐scan for other three‐membered rings such as C_3_H_3_^+^, Si_3_H_3_^+^ and Si_2_BH are shown in SI (Figure S18).

**Figure 3:**
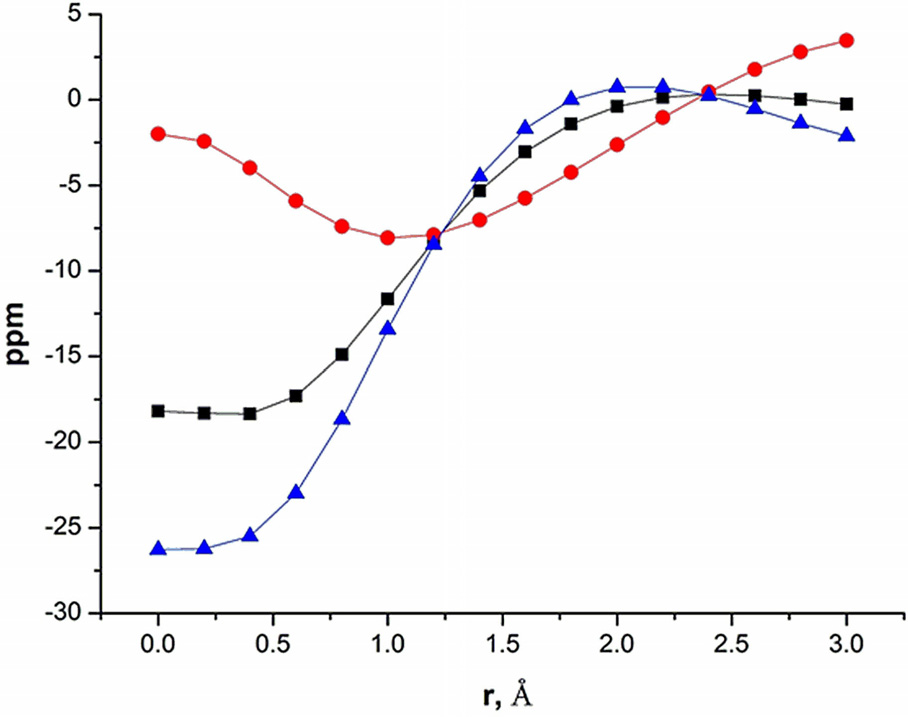
NICS‐scan curves for the three‐membered Si_2_B ring of Disilaborirane, **1**: (▪) isotropic NICS; (•) out‐of‐plane component; (▴) in‐plane component.

The inherent strain and reactivity anticipated for three‐membered rings and the frontier orbitals of **1** suggest several possible reactions.^[19]^ Nitrene, with precedence of adding to cyclopropane to give azacyclobutane, is an attractive reagent in this context.^[19e]^ Accordingly, trimethylsilylnitrene is generated from the corresponding azide in toluene solution of **1** at room temperature (Scheme 2). The solvent is reduced to 5 mL under vacuum and green block‐shaped crystals of four‐membered heterocycle (**2**) (1‐aza‐2,3‐disila‐4‐boretidine derivative)^[20]^ are obtained in 78 % yield at −30 °C after two days. Compound **2** is fully characterized by NMR (^1^H, ^13^C, ^11^B, ^29^Si, Figures S7–S10, SI) and mass spectrometry (LIFDI, Figure S11, SI) methods. The ^11^B NMR spectrum of **2** exhibits a sharp singlet at *δ*=−31.18 ppm and shifts to downfield with respect to **1** whereas the ^29^Si NMR resonances are observed at *δ*=−9.55 ppm for NSiMe_3_ and *δ*=−31.94 ppm for amidinato‐Si.

**Scheme 2:**
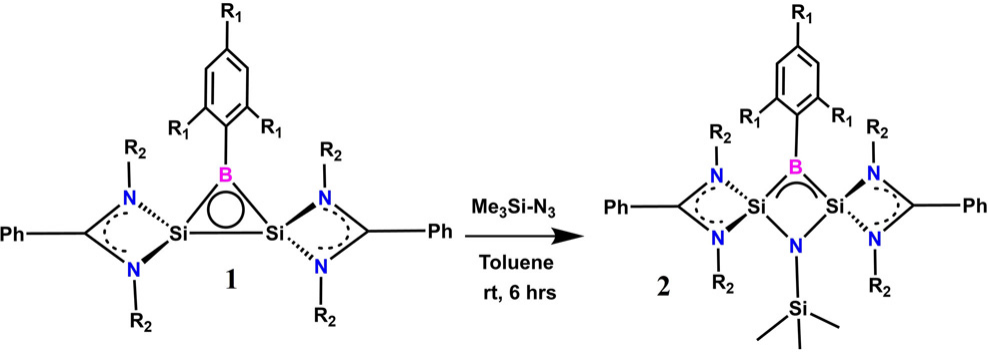
Synthetic scheme for the preparation of **2** (R_1_=isopropyl; R_2_=tert‐butyl).

The molecular structure is unambiguously confirmed by single‐crystal X‐ray diffraction studies (Figure 4). Compound **2** crystallizes in the monoclinic space group *P*2_1_/*n* with one molecule of **2** and one and a half toluene molecules in the asymmetric unit. As expected, compound **2** shows a planar four‐membered Si1‐B1‐Si2‐N1 heterocycle, resulting from the insertion of the nitrene in the Si‐Si sigma bond. The two Si‐B distances are nearly identical Si1/2‐B1 (1.9211(18) and 1.9205(18) Å) and so are the Si1/2‐N1 (1.7633(13) and 1.7687(13) Å) bond lengths. The amidinato‐ligands coordinate to the Si atoms in the same fashion and with similar bond lengths as in **1**. These Si‐N sigma bonds are 0.13 Å longer on the average than the Si‐N distance in compounds with the same ligands where the σ* MOs of π symmetry are vacant.^[16]^

**Figure 4:**
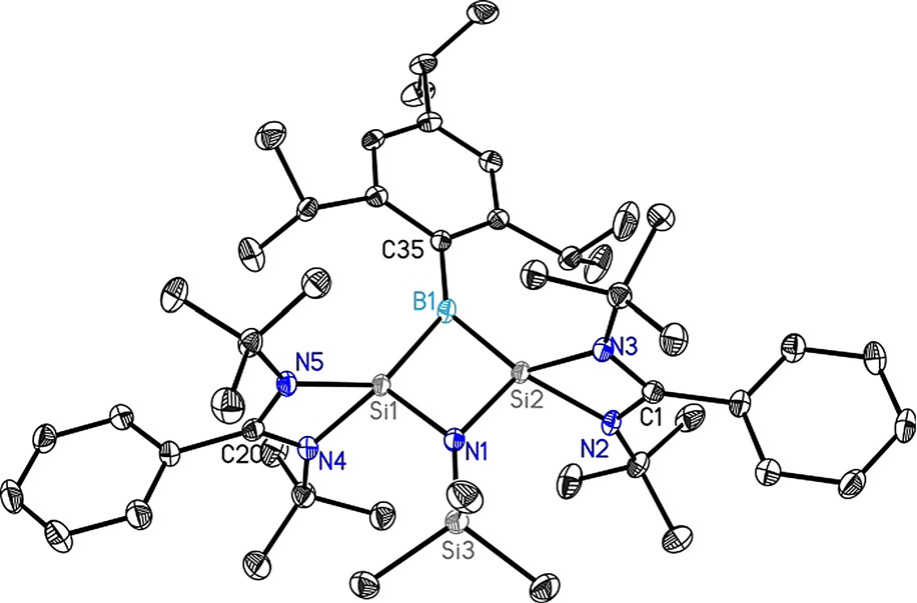
Molecular structure of **2** with anisotropic displacement parameters at the 50 % probability level. Hydrogen atoms are omitted for clarity. Selected bond lengths [Å] and angles of **2** [°]; Si1⋅⋅⋅Si2 2.4367(7), Si1–B1 1.9211(18), Si2–B1 1.9205(18), Si1–N1 1.7633(13), Si2–N1 1.7687(13), Si1–N4 1.8749(14), Si1–N5 1.8921(14), Si2–N2 1.8896(13), Si2–N3 1.8761(14), B1⋅⋅⋅N1 2.763; Si1‐B1‐Si2 78.73(7), B1‐Si2‐N1 96.93(7), Si2‐N1‐Si1 87.24(6), N1‐Si1‐B1 97.09(7).

The aromaticity is disturbed when compound **1** is converted to **2**. The HOMO of **2** is an antibonding combination of the HOMO of **1** with a small negative contribution from the p orbital on NSiMe_3_ (Figure 5 a). Corresponding bonding combination is HOMO‐6 of **2** (Figure 5 b) which is largely localized on NSiMe_3_ (Table S10, SI). With these two π MOs filled, the four‐membered ring is not aromatic. A noticeable point in **2**, in relation to **1**, is that the Si‐Si distance is only stretched by a small amount 0.248 Å to 2.436 Å (crystal structure) in **2**, which is within the range of single bond length. However, NBO analysis does not support a Si−Si bond (Table S6, SI). The Si‐B σ and π bond orders (BOs) remain similar in **1** and **2** (*σ* 0.943 vs. 0.896, π 0.345 vs. 0.301). Newly formed Si−N bonds have predominant σ character (*σ* 0.523 and π 0.067). The Si‐Si BO decreases dramatically (*σ* 0.920 in **1** vs. 0.121 in **2**, and π 0.208 in **1** vs. 0.104 in **2**), even though the Si‐Si distance remains in the bonding range.

**Figure 5:**
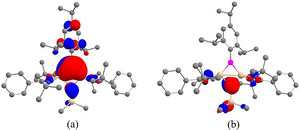
π‐MOs of **2** a) HOMO and b) HOMO−6. Hydrogen atoms are omitted for clarity.

A classical valence bond picture of 2c‐2e *σ* bonds does not give room for a Si‐Si sigma bond in **2**. If there is no σ bond, why is the Si‐Si distance not much elongated? Here comes a situation similar to what is seen in 1,3‐disila‐2,4‐dioxacyclobutane, Si_2_O_2_H_4_ where short Si‐Si distance results from O‐O repulsion than Si−Si bonding.^[21]^ The sum of atomic radii of B and N is 3.3 Å. The non‐bonded B–N distance in **2** is shortened to 2.763 Å already. Any further lengthening of Si‐Si distance would bring the two non‐bonded atoms, boron and nitrogen both of which have partial negative charge, even closer (Table S11, SI). The observed geometry is a balance of all of these.

In conclusion, we have designed, synthesized and fully characterized a neutral 2π aromatic three‐membered disilaborirane, where the aromaticity stems from the delocalization of two σ* MOs on Si and a p orbital on boron. Moreover, we have converted the three‐membered aromatic ring by a unique reaction with (CH_3_)_3_SiN_3_ to a four‐membered non‐aromatic ring. Our approach to involve σ* MOs in delocalization extends the heterogeneity of aromaticity further to an entirely new class of compounds and it is a challenge to continue this research.

## Conflict of interest

The authors declare no conflict of interest.

## Supporting information

As a service to our authors and readers, this journal provides supporting information supplied by the authors. Such materials are peer reviewed and may be re‐organized for online delivery, but are not copy‐edited or typeset. Technical support issues arising from supporting information (other than missing files) should be addressed to the authors.

Supplementary
