# Supplementary material for: A Neutral Three‐Membered 2π Aromatic Disilaborirane and the Unique Conversion into a Four‐Membered BSi2N‐Ring
Source: Angew Chem Int Ed Engl. 2020 Oct 12;59(51):23015–9. doi: 10.1002/anie.202009638 (PMC7756765; doi:10.1002/anie.202009638)
Supplement: Supplementary file 1 — Supplementary [file ANIE-59-23015-s001.pdf]

Angewandte Chemie
Eine Zeitschrift der Gesellschaft Deutscher Chemiker

Supporting Information

A Neutral Three-Membered 2π Aromatic Disilaborirane and the Unique Conversion into a Four-Membered BSi_2N-Ring

Samir Kumar Sarkar, Rinkumoni Chaliha, Mujahuddin M. Siddiqui, Samya Banerjee, Annika Münch, Regine Herbst-Irmer, Dietmar Stalke, Eluvathingal D. Jemmis,* and Herbert W. Roesky**

anie_202009638_sm_miscellaneous_information.pdf

Supporting Information

Table of Contents: **Page Number**

(S1) Materials and Methods (2-3)

(S2) NMR, Mass and UV spectra (4-10)

(S3) X-Ray Crystallographic Analysis (11-21)

(S4) Theoretical calculations (22-29)

(S5) Cartesian coordinates (30-34)

(S6) References (35)

(S1) Materials and Methods

General considerations

All manipulations are carried out using standard Schlenk and glovebox techniques under high purity dinitrogen gas atmosphere. Hexane, toluene and THF are distilled over Na/K alloy (25:75). Deuterated NMR solvent C_6D_6 is dried by stirring for 2 days over Na/K alloy followed by distillation in vacuum and degassed. ^{1}H, ^{13}C, ^{11}B and ^{29}Si NMR spectra are recorded on Bruker Avance 300 and 500 MHz NMR spectrometers and referenced to the resonances of the solvent used. Microanalyses are performed by the Analytisches Labor für Anorganische Chemie at the Universität Göttingen. Melting points are determined in sealed glass capillaries under dinitrogen gas atmosphere. LIFDI measurements are performed on a Joel AccuTOF spectrometer under an inert atmosphere. The starting material dibromo(2,4,6-triisopropylphenyl)borane and amidinato-silylene chloride [LSi-Cl] (L = PhC(NtBu)$_2$),[1] are synthesized by following literature procedures, while all other reagents are used as received.

Synthesis of three-membered disilaborirane ring (1):

A mixture of amidinato-silylene chloride [LSi-Cl] (L = PhC(NtBu)$_2$) (294 mg, 1.0 mmol), dibromo(2,4,6-triisopropylphenyl)borane (188 mg, 0.5 mmol) and KC_8 (270 mg, 2 mmol) are placed in a 100 mL round bottom flask and 40 mL of THF is added at –78 °C. The reaction mixture is slowly warmed to room temperature to get a red solution of compound **1** after 24 hours of stirring. THF is removed under high vacuum and 50 mL of dry toluene is added and stirred for one hour. After filtration of insoluble residue, the solvent is concentrated to 5 mL under high vacuum. The red solution is stored at –30 °C freezer for three days to get X-ray quality red block-shaped crystals of **1** (Yield: 194 mg, 53 %). ^{1}H NMR (500 MHz, C_6D_6): δ = 7.35 (s, 2H, Ar-CH), 6.86 (m, *J* = 5.0 Hz, 10H, Ar-CH), 5.10 (m, *J*= 10 Hz, 1H), 3.01 (m, *J* = 10 Hz, 2H), 1.75 (d, *J*= 10 Hz, 12H), 1.39 (d, *J* = 5 Hz, 6H), 1.38 (s, 36H); ^{13}C NMR (126 MHz, C_6D_6): δ = 171.6 (NCN), 151.1 (Ar-C), 143.6 (Ar-C), 134.2 (Ar-C), 128.6 (Ar-C), 128.4 (Ar-C), 128.2 (Ar-C), 128.0 (Ar-C), 127.9 (Ar-C), 127.7 (Ar-C), 127.4 (Ar-C), 119.3 (Ar-C), 54.5 ($_t$Butyl-C), 35.1 (Isobutyl-C), 33.7 (CH_3), 32.5 (CH_3), 25.3 (CH_3), 24.8 (CH_3); ^{29}Si NMR (99 MHz, 298 K, C_6D_6, ppm): δ = −71.03, ^{11}B NMR (160 MHz, 298 K, C_6D_6): δ = 11.09. Exact Mass: 732.52, Found: 732.50; Melting range:

219-223 °C to a red-orange colored liquid; Anal (%). calcd for $C_{45}H_{69}BN_4Si_2$ (732.52): C, 73.73; H, 9.49; N, 7.64 Found: C, 73.42; H, 10.18; N, 6.73.

Synthesis of four-membered heterocycle (**2**):

A mixture of **1** (60 mg, 0.1 mmol) is taken in a 100 mL round bottom flask and 20 mL of toluene is added at room temperature. Then trimethylsilyl azide ($(CH_3)_3SiN_3$) (87 mg, 0.1 mmol) is added by a syringe slowly. The reaction mixture is stirred at room temperature for six hours. After filtration under inert atmosphere, the solvent is concentrated to 5 mL under high vacuum. The dark green solution is stored at –30 °C in a freezer for two days to get X-ray quality green block-shaped crystals of **2** (Yield: 73 mg, 78 %). ^{1}H NMR (300 MHz, C_6D_6): δ = 7.52 (m, *J* = 3.0 Hz, 2H, Ar-CH), 7.40 (m, *J* = 3.0 Hz, 2H, Ar-CH), 7.27 (s, 2H), 6.95 (m, *J*= 3.0 Hz, 6H, Ar-CH), 4.64 (m, *J* = 6.0 Hz, 2H), 3.07 (m, *J* = 6.0 Hz, 1H), 1.74 (d, *J* = 6.0 Hz, 12H), 1.45 (d, *J* = 6.0 Hz, 6H), 1.38 (s, 36H), 0.57 (s, 9H); ^{13}C NMR (75 MHz, C_6D_6): δ = 173.3 (NCN), 149.1 (Ar-C), 138.3 (Ar-C), 132.4 (Ar-C), 129.4 (Ar-C), 128.5 (Ar-C), 128.1 (Ar-C), 119.3 (Ar-C), 54.2 ($_t$Butyl-C), 34.4 (Isobutyl-C), 33.9 (CH_3), 31.2 (CH_3), 25.9 (CH_3), 24.6 (CH_3), 4.8 (CH_3); ^{29}Si{1H} NMR (99 MHz, 298 K, C_6D_6, ppm) δ = −9.55, −31.94; ^{11}B NMR (160 MHz, 298 K, C_6D_6): δ = −31.18. Exact Mass: 820.25, Found: 820.30; Melting range: 211-214 °C to a green color liquid; Anal (%). calcd for $C_{48}H_{78}BN_5Si_3$ (820.25): C, 70.29; H, 9.59; N, 8.54 Found: C, 63.25; H, 9.17; N, 8.24 (compound **2** is not stable at the experimental conditions).

(S2) NMR, Mass and UV spectra:

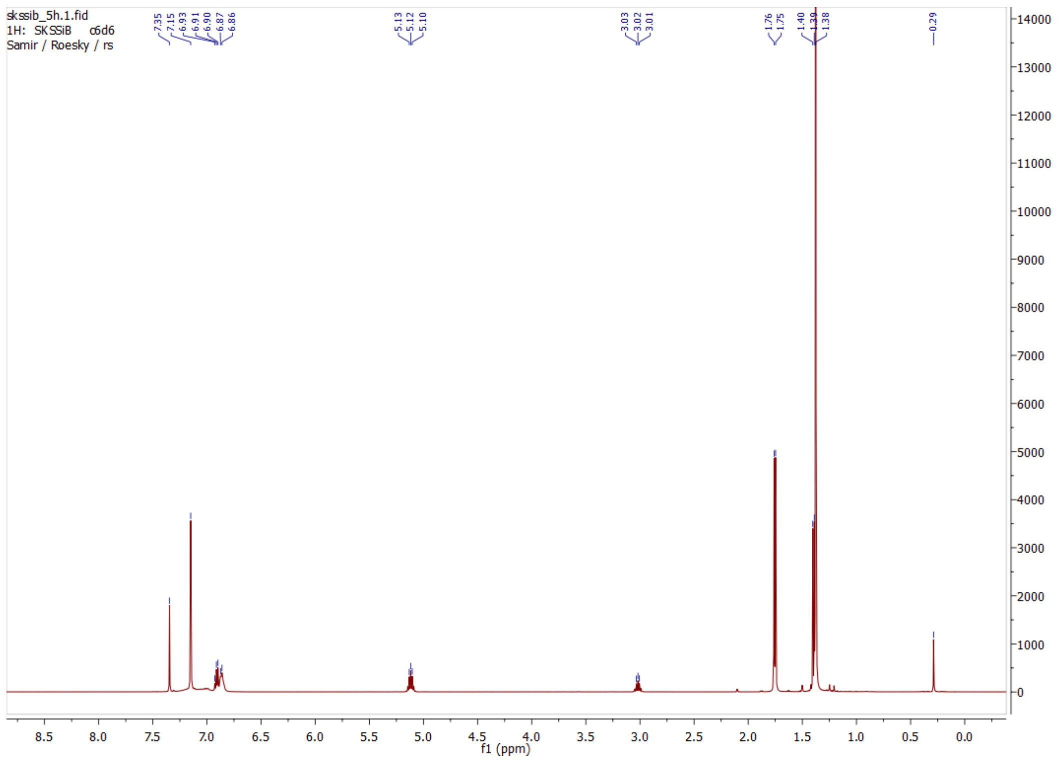

Fig. S1. ^{1}H-NMR spectrum of **1**.

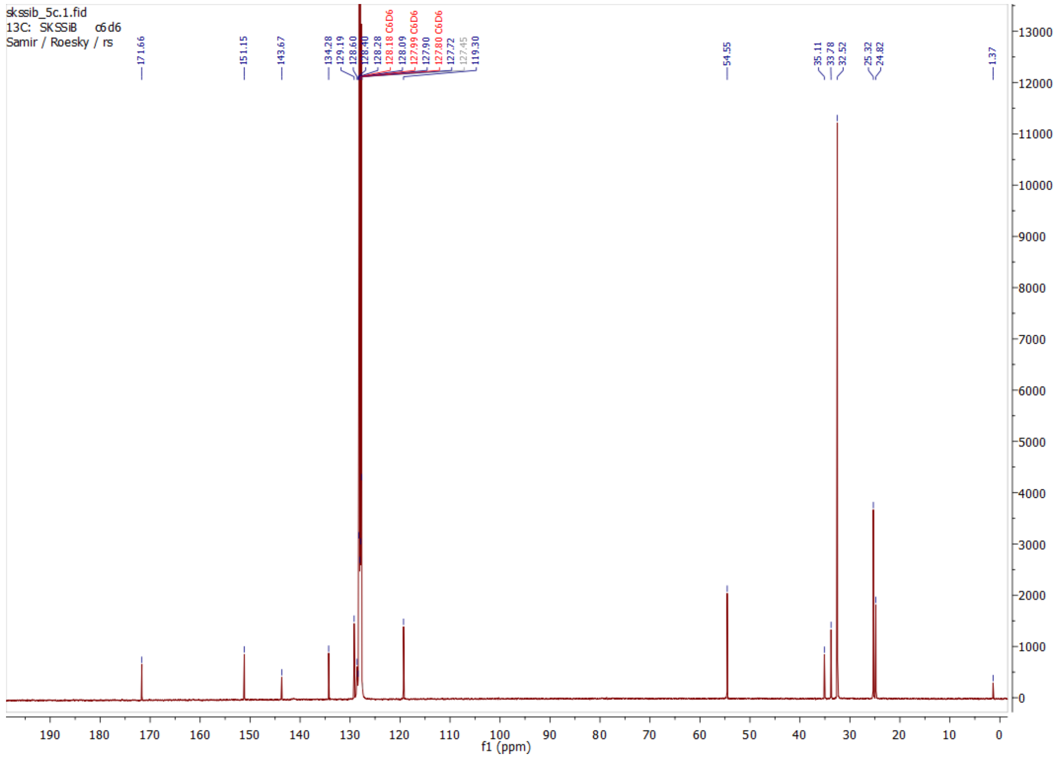

Fig. S2. ^{13}C-NMR spectrum of **1**.

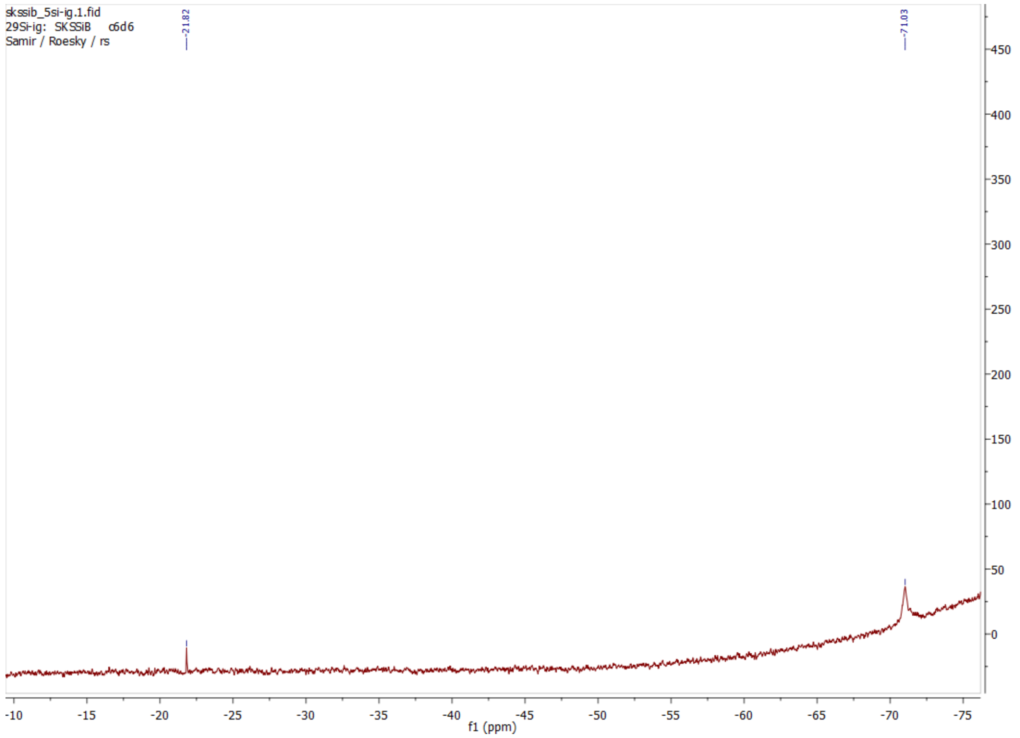

Fig. S3. ^{29}Si-NMR spectrum of **1** (grease peaks at -21.82 ppm).

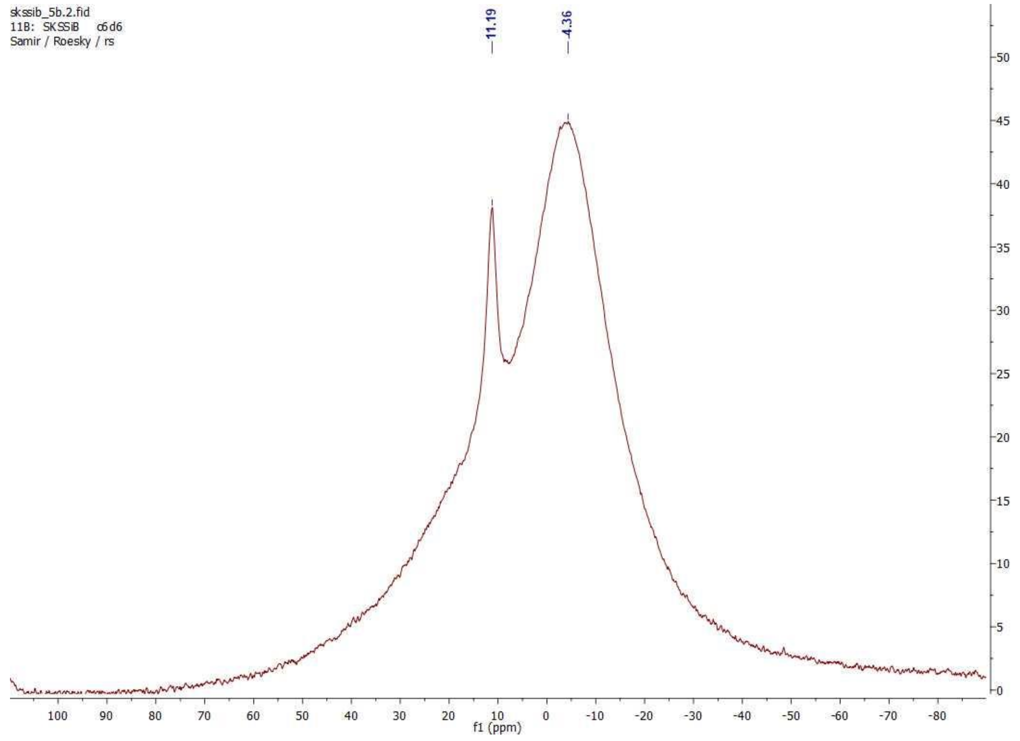

Figure S4. ^{11}B-NMR spectrum of **1**.

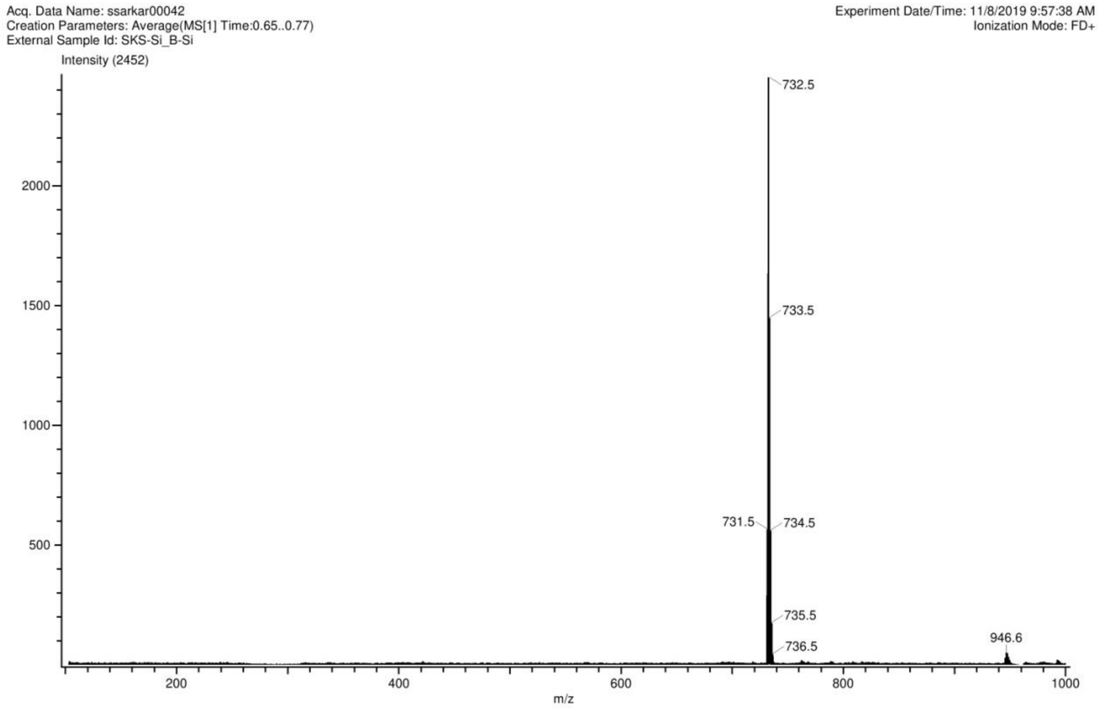

Fig. S5. Mass spectrum of **1**.

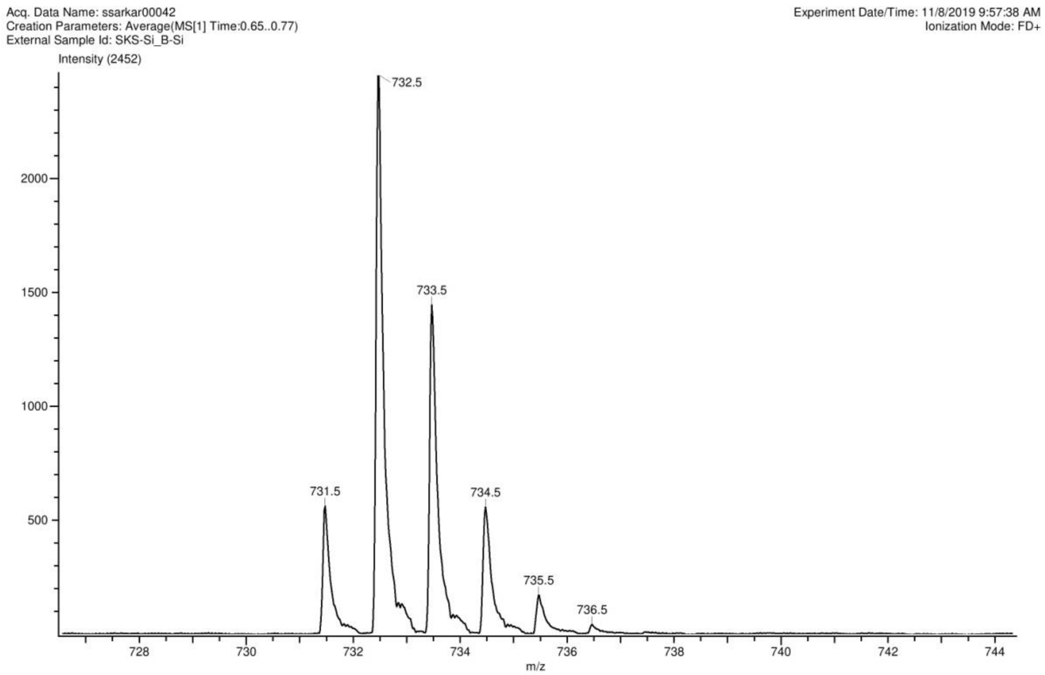

Fig. S6. Isotopic mass distribution spectrum of **1**.

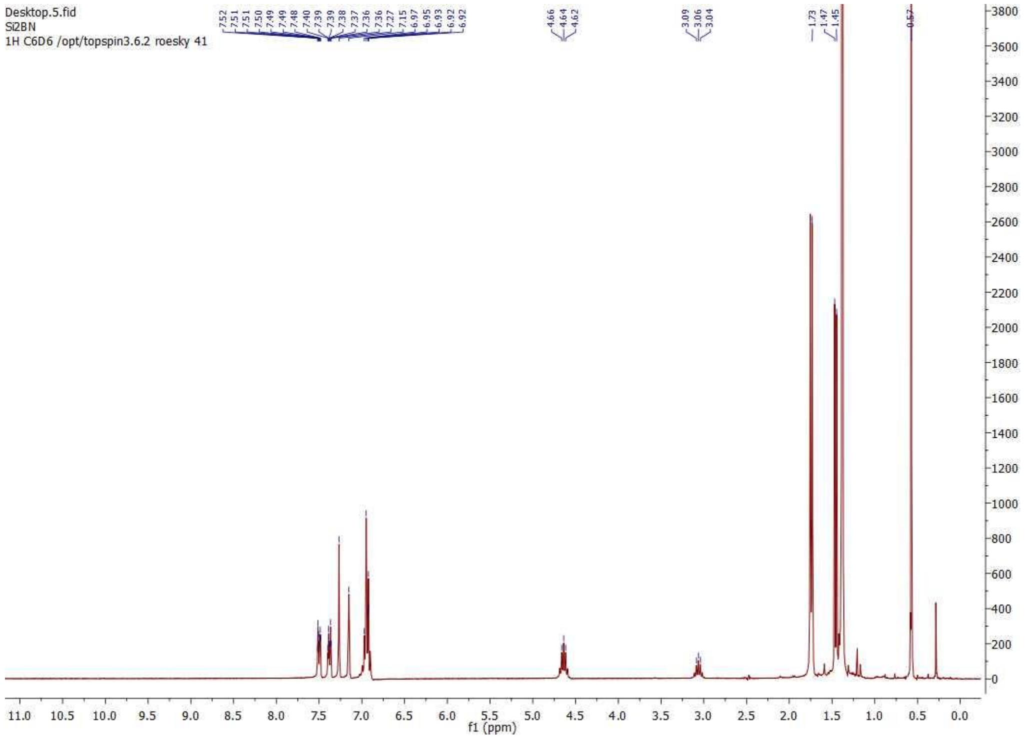

Fig. S7. ^{1}H-NMR spectrum of **2**.

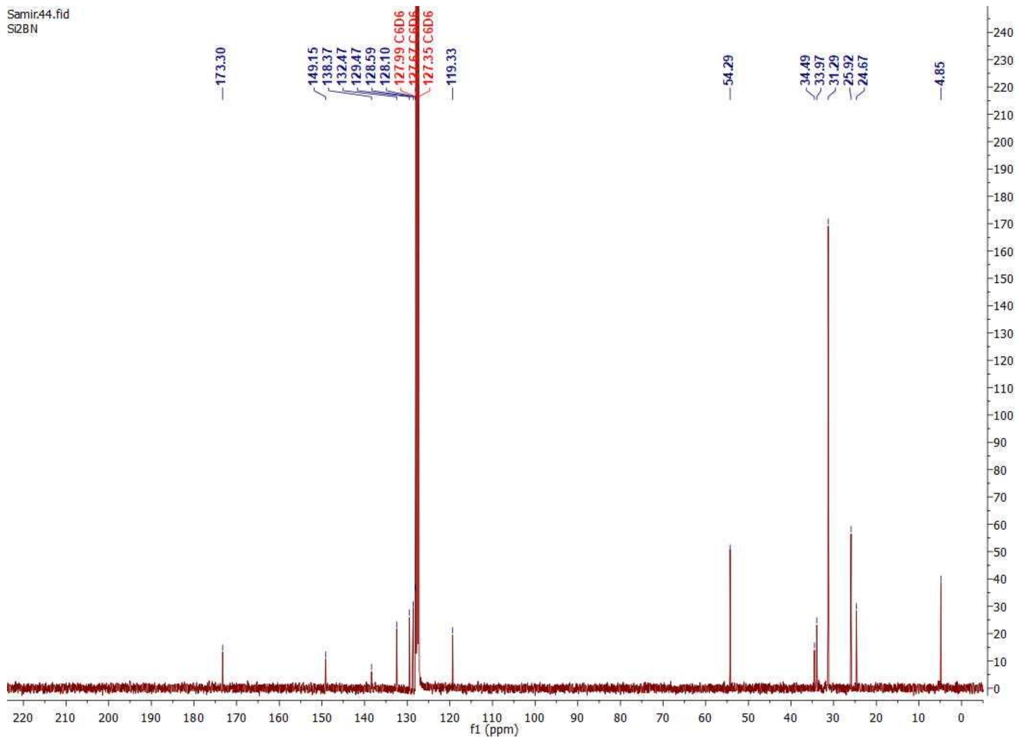

Fig. S8. ^{13}C-NMR spectrum of 2.

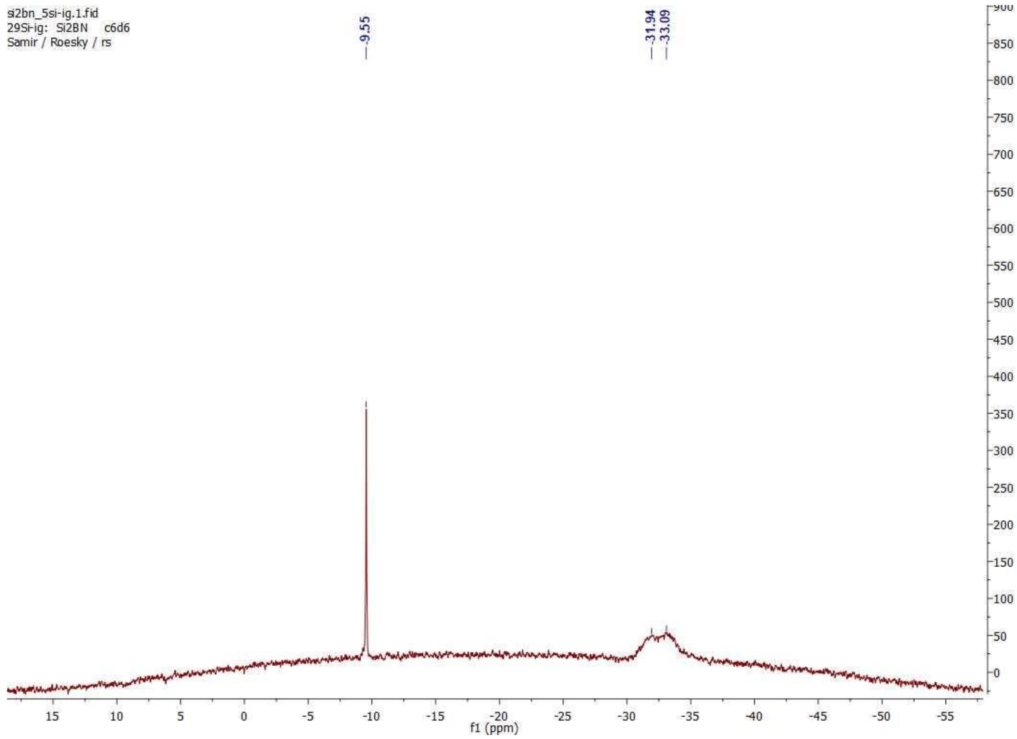

Fig. S9. ^{29}Si-NMR spectrum of **2**.

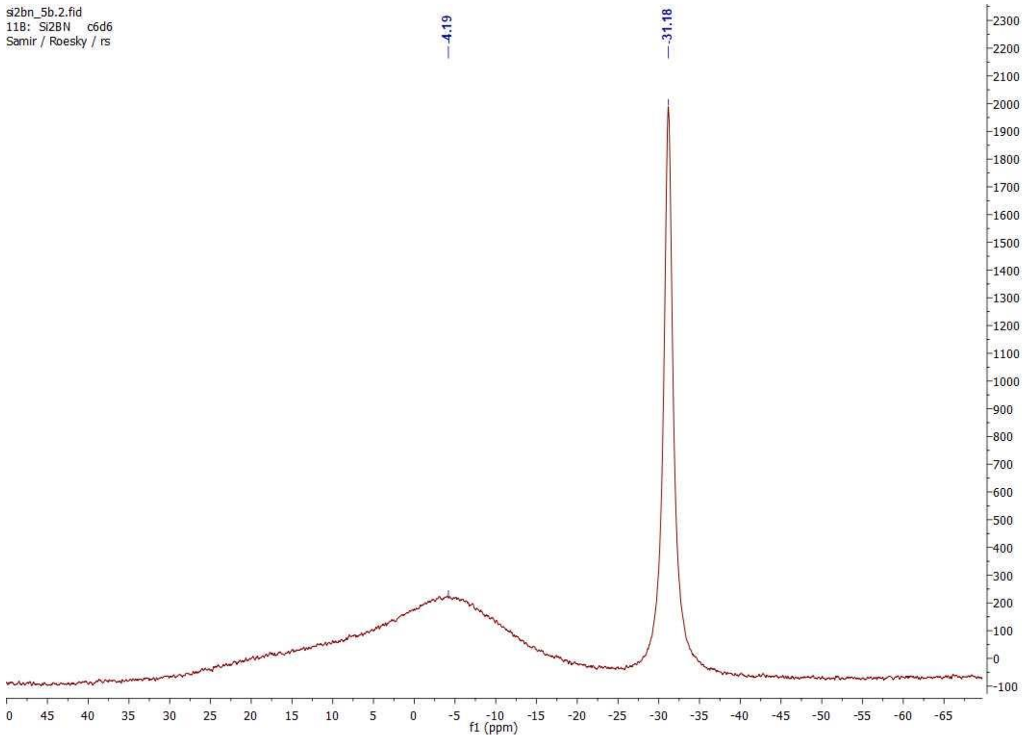

Fig. S10. ^{11}B-NMR spectrum of **2**.

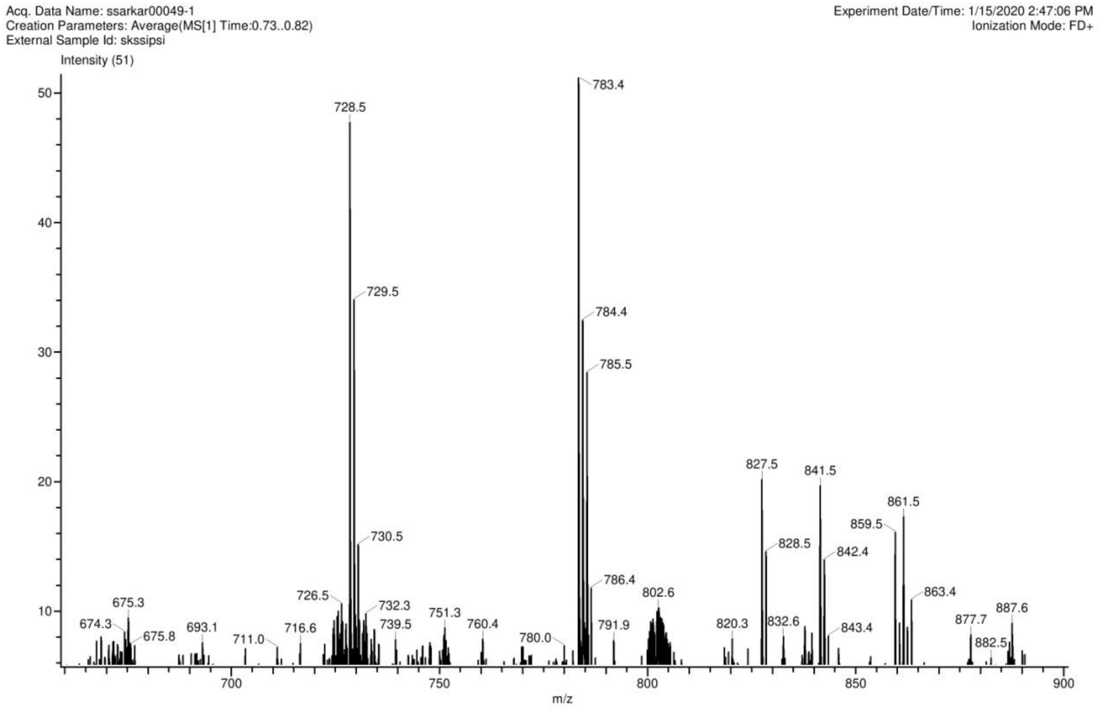

Fig. S11. LIFDI-Mass spectrum of **2**.

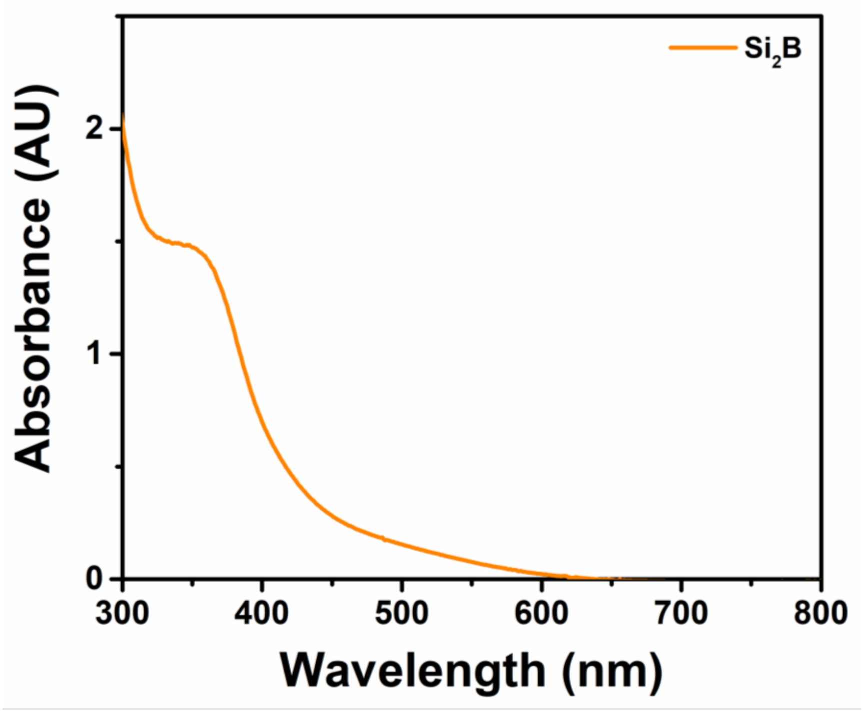

Fig. S12. UV-Vis spectrum of **1** in toluene.

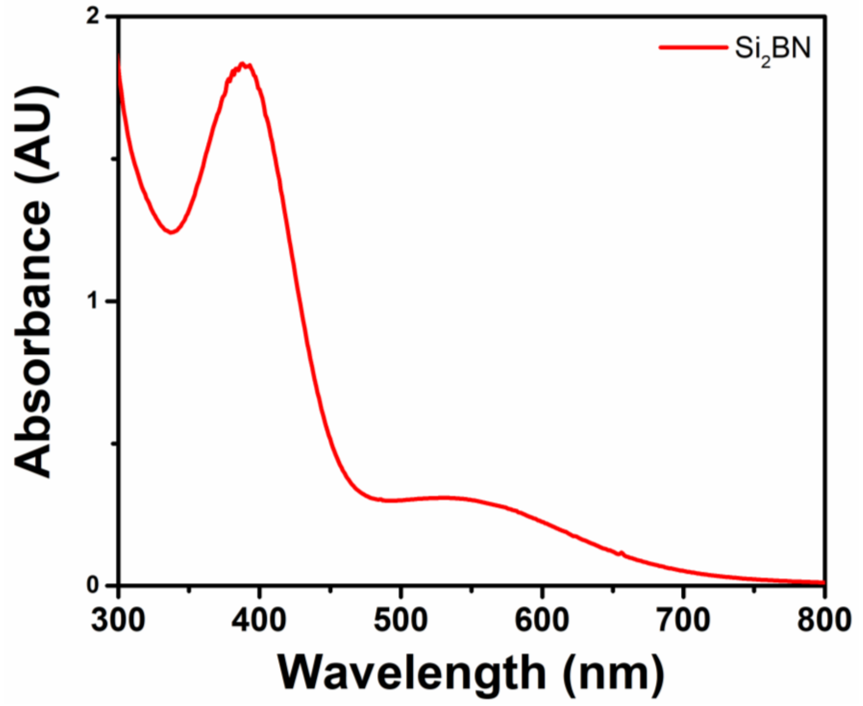

Fig. S13. UV-Vis spectrum of **2** in toluene (λ_{max} = 389 nm and 500 nm).

(S3) X-Ray Crystallographic Analysis: The datasets are collected on an Incoatec Mo Microsource[2] (1, 2) with mirror optics and an APEX II detector with a D8 goniometer. The data are integrated with SAINT.[3] A multi-scan absorption correction and 3λ[4] correction is applied using SADABS.[5] The structures are solved by SHELXT[6] and refined on F^2 using SHELXL[7] in the graphical user interface ShelXle.[8] For the refinement of disordered moieties, distance restraints and restraints for the anisotropic displacement parameters are used.[9]

Table S1. Crystallographic details at 100(2) K.

Compound	**1**	**2**
Formula	$C_{55.50}H_{81}BN_4Si_2$	$C_{58.50}H_{90}BN_5Si_3$
Mol. w., g mol^{-1}	871.23	958.43
CCDC no.	1991565	1991566
Wavelength, Å	0.71073	0.71073
Crystal system	monoclinic	monoclinic
Space group	$P2_1/c$	$P2_1/n$
a, Å	13.832(2)	16.058(3)
b, Å	15.313(3)	13.824(2)
c, Å	25.031(3)	26.418(3)
β	95.86(2)	95.56(2)
V, Å^3	5274.1(14)	5836.8(15)
Z	4	4
Density Mg/m^3	1.097	1.091
Absorption coefficient, mm^{-1}	0.106	0.121
Crystal size, mm	0.331 x 0.311 x 0.208	0.215 x 0.087 x 0.080
Theta range,°	1.48 to 26.39	1.43 to 25.07
Refl. measured	145086	163478
Refl. unique	10795	10336
R_{int}	0.0337	0.0746
Data/ restr./para.	10795 / 1046 / 748	10336 / 188 / 622
R_1 [I>2σ(I)]	0.0340	0.0353
wR_2 (all refl.)	0.0924	0.0908
Extinction coefficient	-	0.00042(10)
$\Delta\rho_{fin}$, eÅ^{-3}	0.363 / -0.248	0.245 / -0.256

Crystal structure of **1**

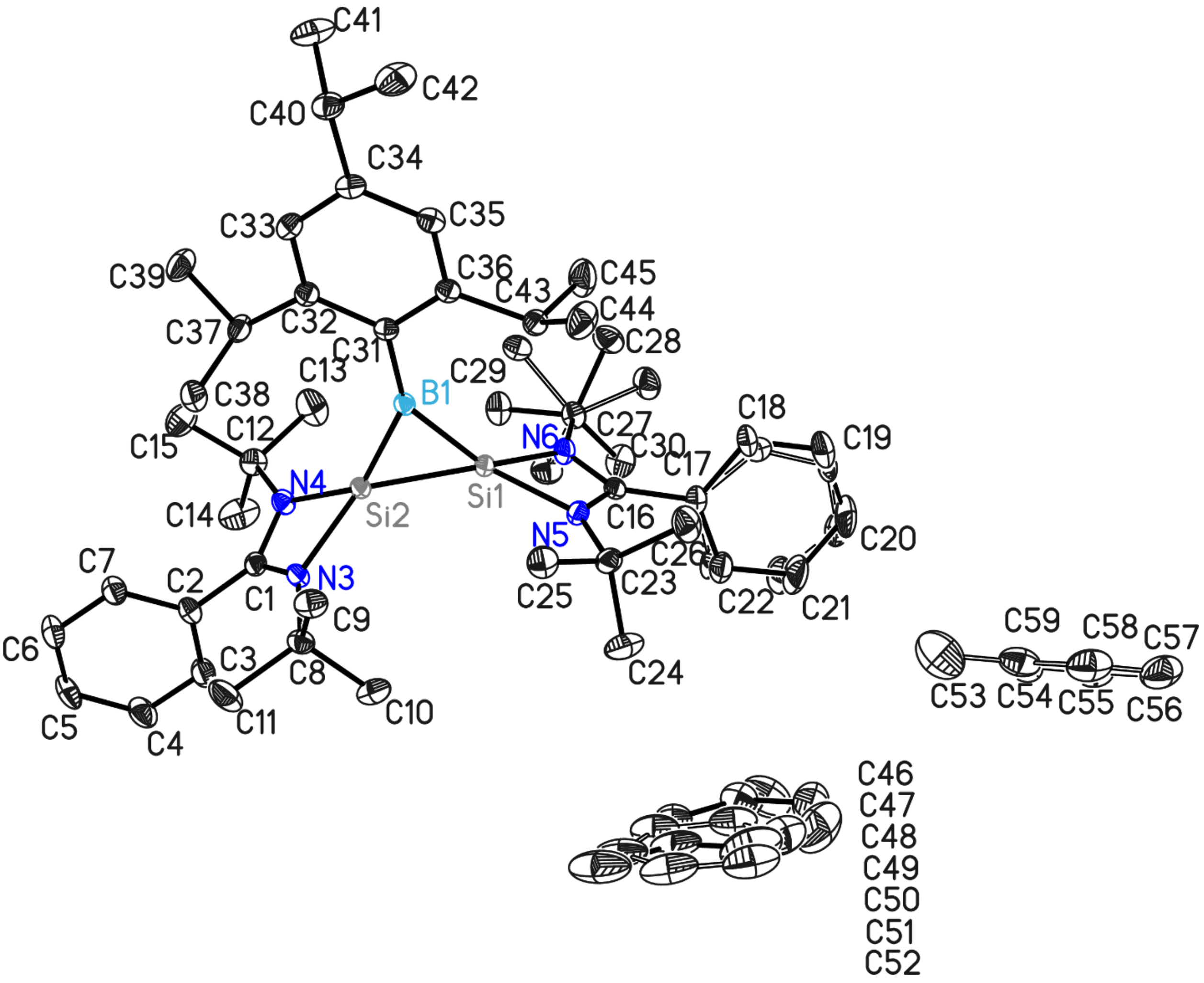

Fig. S14. Crystal structure of **1** with thermal ellipsoids at 50% probability level. The hydrogen atoms are omitted for clarity.

One phenyl ring, a methyl group and both toluene molecules are disordered. The occupancy of the main position of the phenyl ring is refined to 0.743(19) and of the methyl group to 0.827(2). One

toluene molecule is disordered about two positions. The occupancy of the major components is refined to 0.819(3). The second toluene is disordered about the inversion center.

Table S2. Bond lengths [Å] and angles [°] for **1**

.

Si(1)-N(6)	1.8909(10)	C(16)-N(6)	1.3344(15)
Si(1)-N(5)	1.8921(10)	C(16)-C(17)	1.4877(15)
Si(1)-B(1)	1.9190(13)	C(17)-C(22)	1.391(4)
Si(1)-Si(2)	2.1877(5)	C(17)-C(18)	1.397(4)
Si(1)-C(16)	2.3537(12)	C(17)-C(18')	1.402(12)
C(1)-N(3)	1.3262(15)	C(17)-C(22')	1.415(12)
C(1)-N(4)	1.3406(15)	C(18)-C(19)	1.389(4)
C(1)-C(2)	1.4878(15)	C(19)-C(20)	1.387(4)
C(1)-Si(2)	2.3484(12)	C(20)-C(21)	1.390(5)
B(1)-C(31)	1.5851(15)	C(21)-C(22)	1.389(5)
B(1)-Si(2)	1.9186(13)	C(18')-C(19')	1.385(12)
Si(2)-N(3)	1.8834(10)	C(19')-C(20')	1.349(10)
Si(2)-N(4)	1.8961(10)	C(20')-C(21')	1.345(11)
C(2)-C(3)	1.3906(17)	C(21')-C(22')	1.382(12)
C(2)-C(7)	1.3907(17)	C(23)-C(25)	1.5272(17)
N(3)-C(8)	1.4836(14)	C(23)-C(26)	1.5288(17)
C(3)-C(4)	1.3903(17)	C(23)-C(24)	1.5303(17)
N(4)-C(12)	1.4865(15)	N(6)-C(27)	1.4803(14)
C(4)-C(5)	1.3860(19)	C(27)-C(30')	1.499(6)
N(5)-C(16)	1.3338(15)	C(27)-C(30)	1.5130(18)
N(5)-C(23)	1.4846(14)	C(27)-C(29')	1.513(6)
C(5)-C(6)	1.3851(19)	C(27)-C(28)	1.5207(19)
C(6)-C(7)	1.3884(17)	C(27)-C(29)	1.5495(18)
C(8)-C(9)	1.5296(17)	C(27)-C(28')	1.601(6)
C(8)-C(10)	1.5312(17)	C(31)-C(36)	1.4222(15)
C(8)-C(11)	1.5330(17)	C(31)-C(32)	1.4304(15)
C(12)-C(13)	1.5210(18)	C(32)-C(33)	1.3927(15)
C(12)-C(15)	1.5269(18)	C(32)-C(37)	1.5242(15)
C(12)-C(14)	1.5326(17)	C(33)-C(34)	1.3923(16)

C(34)-C(35)	1.3883(16)
C(34)-C(40)	1.5250(15)
C(35)-C(36)	1.3983(15)
C(36)-C(43)	1.5246(15)
C(37)-C(38)	1.5294(18)
C(37)-C(39)	1.5356(17)
C(40)-C(42)	1.5235(18)
C(40)-C(41)	1.5313(18)
C(43)-C(45)	1.5241(18)
C(43)-C(44)	1.5300(16)
C(46)-C(47)	1.504(3)
C(47)-C(48)	1.387(3)
C(47)-C(52)	1.388(4)
C(48)-C(49)	1.384(3)
C(49)-C(50)	1.377(3)
C(50)-C(51)	1.371(4)
C(51)-C(52)	1.377(5)
C(46')-C(47')	1.479(11)
C(47')-C(52')	1.368(11)
C(47')-C(48')	1.388(11)
C(48')-C(49')	1.357(11)
C(49')-C(50')	1.358(12)
C(50')-C(51')	1.374(11)
C(51')-C(52')	1.378(11)
C(53)-C(54)	1.507(5)
C(54)-C(59)	1.375(7)
C(54)-C(55)	1.390(7)
C(55)-C(56)	1.380(6)
C(56)-C(57)	1.376(7)
C(57)-C(58)	1.387(7)
C(58)-C(59)	1.375(7)
N(6)-Si(1)-N(5)	69.00(4)
N(6)-Si(1)-B(1)	138.36(5)
N(5)-Si(1)-B(1)	132.79(5)

N(6)-Si(1)-Si(2)	135.83(3)
N(5)-Si(1)-Si(2)	140.75(3)
B(1)-Si(1)-Si(2)	55.24(4)
N(6)-Si(1)-C(16)	34.51(4)
N(5)-Si(1)-C(16)	34.49(4)
B(1)-Si(1)-C(16)	149.44(5)
Si(2)-Si(1)-C(16)	155.32(3)
N(3)-C(1)-N(4)	107.08(9)
N(3)-C(1)-C(2)	127.20(10)
N(4)-C(1)-C(2)	125.73(10)
N(3)-C(1)-Si(2)	53.26(6)
N(4)-C(1)-Si(2)	53.82(6)
C(2)-C(1)-Si(2)	179.51(9)
C(31)-B(1)-Si(2)	145.41(8)
C(31)-B(1)-Si(1)	145.02(8)
Si(2)-B(1)-Si(1)	69.51(4)
N(3)-Si(2)-N(4)	69.15(4)
N(3)-Si(2)-B(1)	133.39(5)
N(4)-Si(2)-B(1)	138.67(5)
N(3)-Si(2)-Si(1)	131.87(3)
N(4)-Si(2)-Si(1)	142.96(3)
B(1)-Si(2)-Si(1)	55.25(4)
N(3)-Si(2)-C(1)	34.35(4)
N(4)-Si(2)-C(1)	34.80(4)
B(1)-Si(2)-C(1)	150.82(5)
Si(1)-Si(2)-C(1)	152.79(3)
C(3)-C(2)-C(7)	119.91(11)
C(3)-C(2)-C(1)	120.56(11)
C(7)-C(2)-C(1)	119.53(10)
C(1)-N(3)-C(8)	132.62(9)
C(1)-N(3)-Si(2)	92.39(7)
C(8)-N(3)-Si(2)	134.51(7)
C(4)-C(3)-C(2)	119.60(12)
C(1)-N(4)-C(12)	129.12(10)
C(1)-N(4)-Si(2)	91.38(7)

C(12)-N(4)-Si(2)	138.91(8)	C(22)-C(21)-C(20)	119.8(4)
C(5)-C(4)-C(3)	120.36(12)	C(21)-C(22)-C(17)	119.3(4)
C(16)-N(5)-C(23)	129.01(9)	C(19')-C(18')-C(17)	121.5(11)
C(16)-N(5)-Si(1)	92.05(7)	C(20')-C(19')-C(18')	121.2(11)
C(23)-N(5)-Si(1)	138.93(8)	C(21')-C(20')-C(19')	119.1(11)
C(6)-C(5)-C(4)	120.01(11)	C(20')-C(21')-C(22')	121.8(11)
C(5)-C(6)-C(7)	119.91(12)	C(21')-C(22')-C(17)	120.9(11)
C(6)-C(7)-C(2)	120.15(11)	N(5)-C(23)-C(25)	107.06(9)
N(3)-C(8)-C(9)	106.37(9)	N(5)-C(23)-C(26)	111.86(10)
N(3)-C(8)-C(10)	107.39(9)	C(25)-C(23)-C(26)	108.78(10)
C(9)-C(8)-C(10)	110.66(10)	N(5)-C(23)-C(24)	110.41(10)
N(3)-C(8)-C(11)	114.70(10)	C(25)-C(23)-C(24)	108.42(11)
C(9)-C(8)-C(11)	108.24(10)	C(26)-C(23)-C(24)	110.19(11)
C(10)-C(8)-C(11)	109.45(10)	C(16)-N(6)-C(27)	130.21(10)
N(4)-C(12)-C(13)	106.24(10)	C(16)-N(6)-Si(1)	92.09(7)
N(4)-C(12)-C(15)	111.11(10)	C(27)-N(6)-Si(1)	137.69(8)
C(13)-C(12)-C(15)	108.86(11)	N(6)-C(27)-C(30')	104.4(3)
N(4)-C(12)-C(14)	112.23(10)	N(6)-C(27)-C(30)	106.56(10)
C(13)-C(12)-C(14)	108.99(12)	N(6)-C(27)-C(29')	119.8(3)
C(15)-C(12)-C(14)	109.29(11)	C(30')-C(27)-C(29')	113.2(4)
N(5)-C(16)-N(6)	106.84(9)	N(6)-C(27)-C(28)	109.68(10)
N(5)-C(16)-C(17)	127.24(10)	C(30)-C(27)-C(28)	110.38(12)
N(6)-C(16)-C(17)	125.91(10)	N(6)-C(27)-C(29)	112.26(10)
N(5)-C(16)-Si(1)	53.45(6)	C(30)-C(27)-C(29)	108.29(11)
N(6)-C(16)-Si(1)	53.40(6)	C(28)-C(27)-C(29)	109.63(12)
C(17)-C(16)-Si(1)	178.34(8)	N(6)-C(27)-C(28')	105.8(2)
C(22)-C(17)-C(18)	121.0(3)	C(30')-C(27)-C(28')	107.2(4)
C(18')-C(17)-C(22')	115.0(9)	C(29')-C(27)-C(28')	105.6(4)
C(22)-C(17)-C(16)	121.6(2)	C(36)-C(31)-C(32)	115.96(10)
C(18)-C(17)-C(16)	117.4(3)	C(36)-C(31)-B(1)	121.89(10)
C(18')-C(17)-C(16)	128.4(7)	C(32)-C(31)-B(1)	122.15(10)
C(22')-C(17)-C(16)	115.4(6)	C(33)-C(32)-C(31)	120.87(10)
C(19)-C(18)-C(17)	119.1(4)	C(33)-C(32)-C(37)	118.68(10)
C(20)-C(19)-C(18)	119.9(4)	C(31)-C(32)-C(37)	120.45(10)
C(19)-C(20)-C(21)	120.7(4)	C(34)-C(33)-C(32)	122.51(11)

C(35)-C(34)-C(33)	117.17(10)	C(51)-C(50)-C(49)	118.9(2)
C(35)-C(34)-C(40)	123.20(10)	C(50)-C(51)-C(52)	120.7(3)
C(33)-C(34)-C(40)	119.62(10)	C(51)-C(52)-C(47)	121.4(3)
C(34)-C(35)-C(36)	122.17(10)	C(52')-C(47')-C(48')	116.2(10)
C(35)-C(36)-C(31)	121.26(10)	C(52')-C(47')-C(46')	123.1(10)
C(35)-C(36)-C(43)	117.75(10)	C(48')-C(47')-C(46')	120.7(11)
C(31)-C(36)-C(43)	120.99(10)	C(49')-C(48')-C(47')	121.2(12)
C(32)-C(37)-C(38)	110.74(10)	C(48')-C(49')-C(50')	121.8(12)
C(32)-C(37)-C(39)	113.45(10)	C(49')-C(50')-C(51')	118.6(10)
C(38)-C(37)-C(39)	110.33(10)	C(50')-C(51')-C(52')	119.1(12)
C(42)-C(40)-C(34)	114.19(10)	C(47')-C(52')-C(51')	123.0(12)
C(42)-C(40)-C(41)	109.50(11)	C(59)-C(54)-C(55)	118.2(4)
C(34)-C(40)-C(41)	110.55(10)	C(59)-C(54)-C(53)	120.8(5)
C(45)-C(43)-C(36)	110.99(10)	C(55)-C(54)-C(53)	121.0(5)
C(45)-C(43)-C(44)	110.72(11)	C(56)-C(55)-C(54)	121.3(5)
C(36)-C(43)-C(44)	113.40(10)	C(57)-C(56)-C(55)	119.9(6)
C(48)-C(47)-C(52)	117.4(3)	C(56)-C(57)-C(58)	119.1(5)
C(48)-C(47)-C(46)	121.0(3)	C(59)-C(58)-C(57)	120.6(6)
C(52)-C(47)-C(46)	121.6(3)	C(58)-C(59)-C(54)	120.9(5)
C(49)-C(48)-C(47)	120.9(3)		
C(50)-C(49)-C(48)	120.7(3)		

Crystal structure of **2**

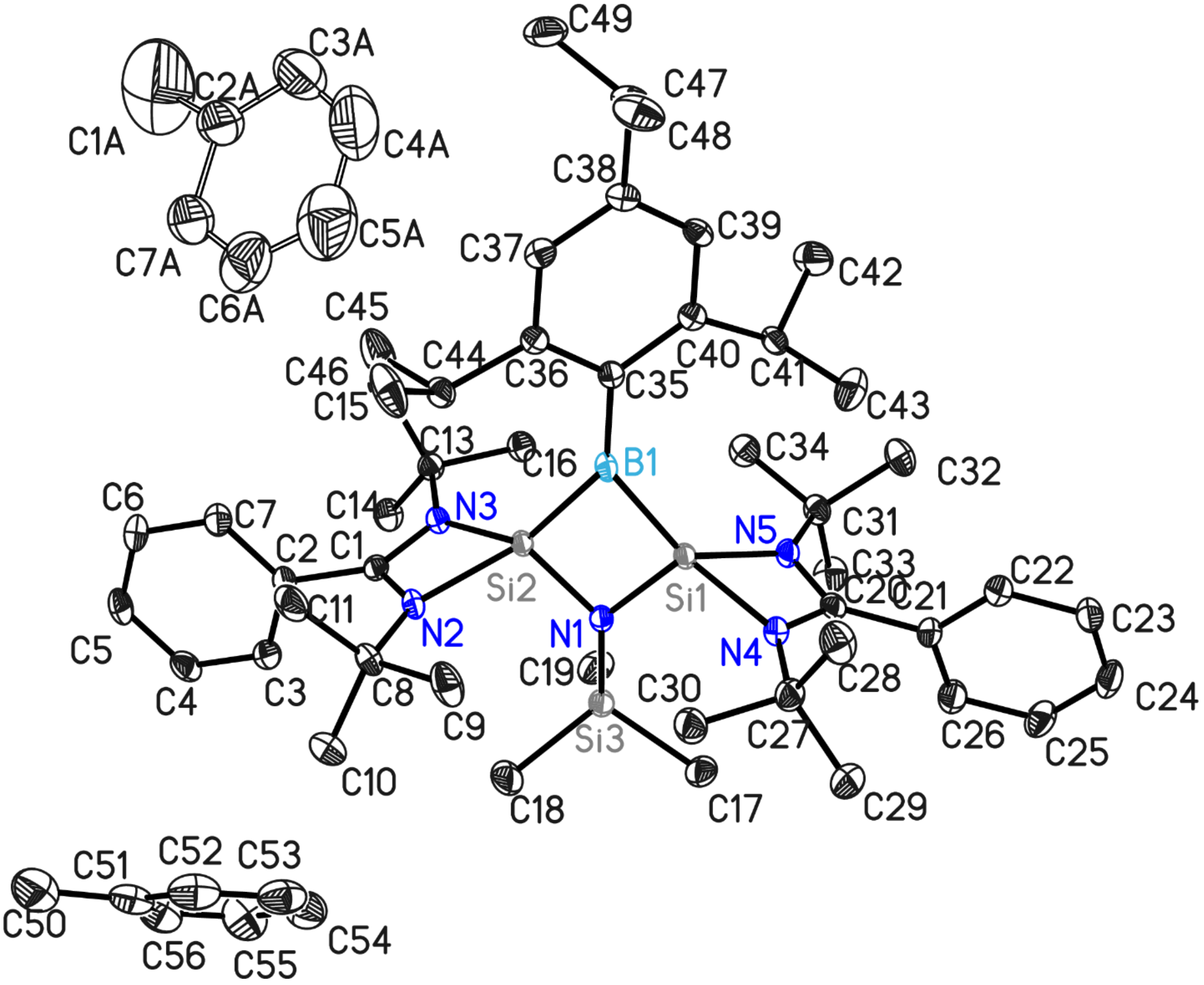

Fig. S15. Crystal structure of **2** with thermal ellipsoids at 50% probability level. The hydrogen atoms are omitted for clarity.

One of the toluene molecule is disordered about the inversion centre.

Table S3. Bond lengths [Å] and angles [°] for **2**.

Si(1)-N(1)	1.7633(13)	C(13)-C(14)	1.530(2)
Si(1)-N(4)	1.8749(14)	C(13)-C(15)	1.531(2)
Si(1)-N(5)	1.8921(14)	C(25)-C(26)	1.384(2)
Si(1)-B(1)	1.9211(18)	C(25)-C(24)	1.386(3)
Si(1)-C(20)	2.3427(17)	C(24)-C(23)	1.384(2)
Si(1)-Si(2)	2.4367(7)	C(23)-C(22)	1.389(2)
B(1)-C(35)	1.589(2)	C(22)-C(21)	1.400(2)
B(1)-Si(2)	1.9205(18)	C(21)-C(26)	1.391(2)
C(1)-N(3)	1.336(2)	C(21)-C(20)	1.491(2)
C(1)-N(2)	1.337(2)	C(28)-C(27)	1.531(2)
C(1)-C(2)	1.491(2)	C(27)-C(30)	1.526(2)
C(1)-Si(2)	2.3401(16)	C(27)-C(29)	1.530(2)
N(1)-Si(3)	1.7174(13)	Si(2)-N(2)	1.8896(13)
N(1)-Si(2)	1.7687(13)	C(31)-C(34)	1.525(2)
Si(3)-C(18)	1.8649(19)	C(31)-C(32)	1.528(2)
Si(3)-C(17)	1.8659(18)	C(31)-C(33)	1.534(2)
Si(3)-C(19)	1.8787(19)	C(35)-C(40)	1.431(2)
C(3)-C(4)	1.384(2)	C(35)-C(36)	1.432(2)
C(3)-C(2)	1.394(2)	C(36)-C(37)	1.397(2)
N(3)-C(13)	1.489(2)	C(36)-C(44)	1.521(2)
N(3)-Si(2)	1.8761(14)	C(37)-C(38)	1.390(2)
N(5)-C(20)	1.338(2)	C(38)-C(39)	1.383(2)
N(5)-C(31)	1.489(2)	C(38)-C(47)	1.517(2)
C(5)-C(4)	1.384(2)	C(39)-C(40)	1.398(2)
C(5)-C(6)	1.388(2)	C(40)-C(41)	1.520(2)
N(4)-C(20)	1.333(2)	C(41)-C(43)	1.520(2)
N(4)-C(27)	1.488(2)	C(41)-C(42)	1.529(2)
C(9)-C(8)	1.524(2)	C(44)-C(45)	1.517(3)
C(8)-N(2)	1.492(2)	C(44)-C(46)	1.525(2)
C(8)-C(10)	1.528(2)	C(47)-C(49)	1.528(2)
C(8)-C(11)	1.531(2)	C(47)-C(48)	1.531(2)
C(7)-C(6)	1.385(2)	C(50)-C(51)	1.504(3)
C(7)-C(2)	1.392(2)	C(51)-C(52)	1.384(3)
C(13)-C(16)	1.524(2)	C(51)-C(56)	1.385(3)

C(52)-C(53)	1.377(3)	C(2)-C(1)-Si(2)	179.17(12)
C(53)-C(54)	1.376(3)	Si(3)-N(1)-Si(1)	135.12(8)
C(54)-C(55)	1.376(3)	Si(3)-N(1)-Si(2)	137.29(8)
C(55)-C(56)	1.380(3)	Si(1)-N(1)-Si(2)	87.24(6)
C(1A)-C(2A)	1.517(9)	N(1)-Si(3)-C(18)	112.10(7)
C(2A)-C(3A)	1.381(9)	N(1)-Si(3)-C(17)	110.93(7)
C(2A)-C(7A)	1.403(9)	C(18)-Si(3)-C(17)	106.52(9)
C(3A)-C(4A)	1.439(11)	N(1)-Si(3)-C(19)	114.53(8)
C(4A)-C(5A)	1.351(11)	C(18)-Si(3)-C(19)	105.25(9)
C(5A)-C(6A)	1.303(8)	C(17)-Si(3)-C(19)	107.00(8)
C(6A)-C(7A)	1.372(9)	C(4)-C(3)-C(2)	119.99(16)
		C(1)-N(3)-C(13)	129.62(13)
N(1)-Si(1)-N(4)	111.66(6)	C(1)-N(3)-Si(2)	91.97(10)
N(1)-Si(1)-N(5)	110.49(6)	C(13)-N(3)-Si(2)	138.09(10)
N(4)-Si(1)-N(5)	69.38(6)	C(20)-N(5)-C(31)	129.55(13)
N(1)-Si(1)-B(1)	97.09(7)	C(20)-N(5)-Si(1)	91.34(10)
N(4)-Si(1)-B(1)	131.25(7)	C(31)-N(5)-Si(1)	138.91(11)
N(5)-Si(1)-B(1)	135.90(7)	C(4)-C(5)-C(6)	119.88(15)
N(1)-Si(1)-C(20)	117.74(6)	C(20)-N(4)-C(27)	130.49(14)
N(4)-Si(1)-C(20)	34.64(6)	C(20)-N(4)-Si(1)	92.25(10)
N(5)-Si(1)-C(20)	34.82(6)	C(27)-N(4)-Si(1)	137.14(11)
B(1)-Si(1)-C(20)	145.08(7)	C(3)-C(4)-C(5)	120.28(16)
N(1)-Si(1)-Si(2)	46.47(4)	N(2)-C(8)-C(9)	105.79(13)
N(4)-Si(1)-Si(2)	140.29(5)	N(2)-C(8)-C(10)	112.92(13)
N(5)-Si(1)-Si(2)	142.87(5)	C(9)-C(8)-C(10)	108.27(14)
B(1)-Si(1)-Si(2)	50.62(5)	N(2)-C(8)-C(11)	110.78(14)
C(20)-Si(1)-Si(2)	164.09(4)	C(9)-C(8)-C(11)	110.19(14)
C(35)-B(1)-Si(2)	141.53(12)	C(10)-C(8)-C(11)	108.82(14)
C(35)-B(1)-Si(1)	139.72(12)	C(6)-C(7)-C(2)	119.99(15)
Si(2)-B(1)-Si(1)	78.73(7)	C(7)-C(6)-C(5)	120.19(16)
N(3)-C(1)-N(2)	107.02(13)	N(3)-C(13)-C(16)	105.93(12)
N(3)-C(1)-C(2)	126.26(14)	N(3)-C(13)-C(14)	111.99(13)
N(2)-C(1)-C(2)	126.69(14)	C(16)-C(13)-C(14)	108.71(13)
N(3)-C(1)-Si(2)	53.25(8)	N(3)-C(13)-C(15)	110.88(13)
N(2)-C(1)-Si(2)	53.83(8)	C(16)-C(13)-C(15)	109.29(13)

C(14)-C(13)-C(15)	109.91(13)	C(1)-Si(2)-Si(1)	162.07(4)
C(26)-C(25)-C(24)	120.37(17)	C(7)-C(2)-C(3)	119.65(15)
C(23)-C(24)-C(25)	119.92(16)	C(7)-C(2)-C(1)	121.18(14)
C(24)-C(23)-C(22)	120.17(16)	C(3)-C(2)-C(1)	119.13(14)
C(23)-C(22)-C(21)	119.98(16)	C(1)-N(2)-C(8)	128.82(13)
C(26)-C(21)-C(22)	119.39(15)	C(1)-N(2)-Si(2)	91.32(10)
C(26)-C(21)-C(20)	120.21(14)	C(8)-N(2)-Si(2)	138.94(10)
C(22)-C(21)-C(20)	120.40(14)	N(5)-C(31)-C(34)	106.18(13)
N(4)-C(20)-N(5)	106.79(13)	N(5)-C(31)-C(32)	112.42(13)
N(4)-C(20)-C(21)	126.85(15)	C(34)-C(31)-C(32)	109.50(14)
N(5)-C(20)-C(21)	126.36(14)	N(5)-C(31)-C(33)	111.13(14)
N(4)-C(20)-Si(1)	53.10(8)	C(34)-C(31)-C(33)	108.31(14)
N(5)-C(20)-Si(1)	53.85(8)	C(32)-C(31)-C(33)	109.18(14)
C(21)-C(20)-Si(1)	175.94(11)	C(40)-C(35)-C(36)	114.52(13)
N(4)-C(27)-C(30)	105.26(13)	C(40)-C(35)-B(1)	122.18(13)
N(4)-C(27)-C(29)	112.60(14)	C(36)-C(35)-B(1)	123.30(13)
C(30)-C(27)-C(29)	109.20(14)	C(37)-C(36)-C(35)	121.79(14)
N(4)-C(27)-C(28)	110.58(14)	C(37)-C(36)-C(44)	116.79(14)
C(30)-C(27)-C(28)	109.51(14)	C(35)-C(36)-C(44)	121.39(13)
C(29)-C(27)-C(28)	109.58(14)	C(38)-C(37)-C(36)	122.64(15)
C(25)-C(26)-C(21)	120.14(16)	C(39)-C(38)-C(37)	116.41(14)
N(1)-Si(2)-N(3)	109.77(6)	C(39)-C(38)-C(47)	121.76(14)
N(1)-Si(2)-N(2)	110.36(6)	C(37)-C(38)-C(47)	121.83(14)
N(3)-Si(2)-N(2)	69.59(6)	C(38)-C(39)-C(40)	123.02(15)
N(1)-Si(2)-B(1)	96.93(7)	C(39)-C(40)-C(35)	121.57(14)
N(3)-Si(2)-B(1)	133.02(7)	C(39)-C(40)-C(41)	116.74(14)
N(2)-Si(2)-B(1)	135.67(7)	C(35)-C(40)-C(41)	121.69(13)
N(1)-Si(2)-C(1)	115.86(6)	C(40)-C(41)-C(43)	111.21(13)
N(3)-Si(2)-C(1)	34.78(6)	C(40)-C(41)-C(42)	113.23(13)
N(2)-Si(2)-C(1)	34.85(6)	C(43)-C(41)-C(42)	110.36(15)
B(1)-Si(2)-C(1)	147.15(7)	C(45)-C(44)-C(36)	110.00(14)
N(1)-Si(2)-Si(1)	46.29(4)	C(45)-C(44)-C(46)	110.56(16)
N(3)-Si(2)-Si(1)	139.47(5)	C(36)-C(44)-C(46)	113.57(13)
N(2)-Si(2)-Si(1)	142.37(5)	C(38)-C(47)-C(49)	112.30(14)
B(1)-Si(2)-Si(1)	50.64(5)	C(38)-C(47)-C(48)	111.39(13)

C(49)-C(47)-C(48)	110.69(15)	C(3A)-C(2A)-C(7A)	117.4(7)
C(52)-C(51)-C(56)	117.7(2)	C(3A)-C(2A)-C(1A)	129.5(8)
C(52)-C(51)-C(50)	121.0(2)	C(7A)-C(2A)-C(1A)	113.1(7)
C(56)-C(51)-C(50)	121.27(19)	C(2A)-C(3A)-C(4A)	121.0(8)
C(53)-C(52)-C(51)	121.2(2)	C(5A)-C(4A)-C(3A)	119.3(8)
C(54)-C(53)-C(52)	120.5(2)	C(6A)-C(5A)-C(4A)	117.3(9)
C(53)-C(54)-C(55)	119.0(2)	C(5A)-C(6A)-C(7A)	127.7(8)
C(54)-C(55)-C(56)	120.4(2)	C(6A)-C(7A)-C(2A)	116.9(7)
C(55)-C(56)-C(51)	121.17(19)		

(S4) Theoretical Studies:

Calculations are performed using Gaussian 09 (version D) program package.[10] Geometry optimizations for all the structures are carried out employing BP86 functional[11-12] and 6-31G (d,p) basis set. Using the same level of theory, Natural population analyses[13-14] are performed as implemented in the Gaussian 09 suite of programs. To study the decomposition of WBI index[15] a multifunctional wave functional analyzer Multiwfn software[16-17] are used along with Gaussian 09. Nucleus-independent chemical shift (NICS)[18] calculations are accomplished with the GIAO (gauge-independent atomic orbitals) method[19] at B3LYP/6-311G+(d,p) level of theory[20] to probe the extent of π delocalization in structure **1** and **2**. To eliminate the σ contributions, NICS has been calculated 1 Å above the molecular plane (NICS(1), isotropic chemical shift) and the ZZ component of the chemical shift tensor (NICSzz) values are computed at the ring center and 1 Å above the ring center. A more elaborate method to probe aromaticity called NICS-scan developed by Stanger[21] is applied by keeping the ghost atoms (Bq's) above the geometric centers of the three-membered rings with an interval of 0.2 Å each along a trajectory orthogonal to the plane crossing the z axis. We have covered distances ranging from 0.0 Å to 3.0 Å. The isotropic chemical shifts of these ghost atoms are given by the NICS values, which are further divided into in-plane and out-of-plane components. We have considered the ZZ component of the chemical shift tensor (NICSzz) as the out-of-plane component of the NICS and the average of the XX and YY component of the same as the in-plane component. The scans are plotted with NICS values (ppm) in the Y axis with distances r (Å) from the molecular plane along X axis.

Table S4. Comparison of selected structural parameters (bond length, Å and angle, degree) of the three-membered ring in **1** and four-membered ring in **2** from X-ray structure analysis and DFT (BP86/6-31G (d,p)); *The numbers in blue signify the labels in the atoms in the crystal structure of **1** and **2**.

**Parameter**	**Structure 1**		**Structure 2**		**Parameter**	**Structure 1**		**Structure 2**	
Bond length (in Å)	X-ray	DFT	X-ray	DFT	Angle (in degree)	X-ray	DFT	X-ray	DFT
Si_1-Si_2	2.188	2.232	2.436	2.451	Si_1-B_1-Si_2	69.508	70.320	78.717	78.873
Si_1-B_1	1.919	1.938	1.921	1.927	B_1-Si_1-Si_2	55.240	54.835	50.649	50.686
Si_2-B_1	1.919	1.938	1.921	1.931	B_1-Si_2-Si_1	55.251	54.844	50.634	50.442
Si_1-N_1	-	-	1.763	1.802	Si_1-N_1-Si_2	-	-	87.258	85.675
Si_2-N_1	-	-	1.769	1.803	B_1-Si_1-Si_2-N_1	-	-	179.990	179.393

Table S5. % Atomic contributions to the important molecular orbitals of structure **1** at BP86/6-31G (d,p). HOMO is largely based on the two silicon and boron atoms.

**MO**		**Si_1**	**Si_2**	**B_1**	**C_{31}**	**N_3**	**N_4**	**N_5**	**N_6**	**C_1**	**C_{16}**	**OTHERS**
**HOMO**	p	21	21	28	-	2	-	2	-	-	-	-
	d	2	2	-	-	-	-	-	-	-	-	-
**HOMO-1**	p	7	7	16	10	2	2	2	2	-	-	C_{34}=9, C_{36}=6, C_{32}=5
	s	8	7	-	-	-	-	-	-	-	-	-

**HOMO-2**	p	27	27	9	4	-	2	-	2	3	3	-
	s	3	2	3	-	-	-	-	-	-	-	-

*Less than 2% atomic contributions are not considered.

*The numbers in blue signify the labels in the atoms in the crystal structure of **1**

Table S6. Bond lengths and σ (red) and π (green) components of WBI for Si-Si, Si-B, Si-N bonds in the three membered rings in **1** and four-membered ring in **2**.

**Structure**		**1**	**$Si_3H_3^+$**	**H_2SiSiH_2**	**$H_2SiBH_2^{-1}$**	**2**
**Si-Si**	Bond length	2.232	2.219	2.191	-	2.451
	**WBI** (σ+π)	**1.128** (0.920+0.208)	**1.406** (0.970+0.436)	**1.903** (1.076+0.827)	**-**	**0.225** (0.121+0.104)
**Si-B**	Bond length	1.938	-	-	1.883	1.929
	**WBI**(σ+π)	**1.288** (0.943+0.345)	-	**-**	**1.893** (1.035+0.858)	**1.197** (0.896+0.301)
**Si-N**	Bond length	-	-	-	-	1.803
	**WBI**(σ+π)	-		**-**	**-**	**0.590** (0.523+0.067)
**B-N**	Bond length	-	-	-	-	2.811
	**WBI**	-		**-**	**-**	**0.075**

Table S7. Natural Charges on structure 1 based on Natural population Analysis at BP86/6-31G (d,p)

Atom/Fragment	Natural Charge
Si_1	0.938
Si_2	0.937
B_1	-0.676
N_3	-0.682
N_4	-0.684
N_5	-0.682
N_6	-0.685
$Si_1(N_5C_{16}N_6)(C_6H_5)(C(CH_3)_3)_2$	0.503
$Si_2(N_3C_1N_4)(C_6H_5)(C(CH_3)_3)_2$	0.502
$B_1(C_6H_2(C(CH_3)_3)_3)$	-1.005

*The numbers in blue signify the labels in the atoms in the crystal structure of **1**

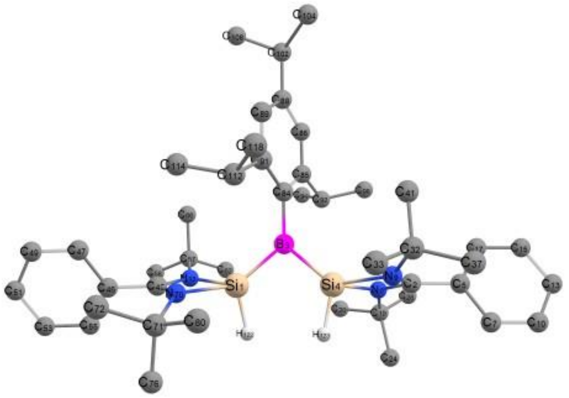

1-H_2 (C_1)

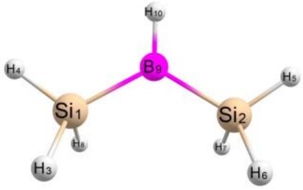

3a (C_{2v})

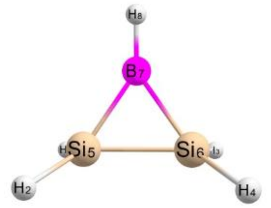

3b (C_{2v})

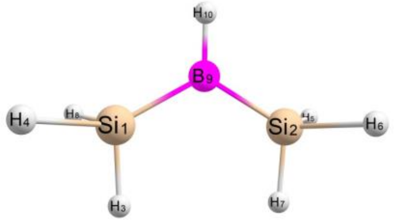

$(3a)^{2-}$ (C_{2v})

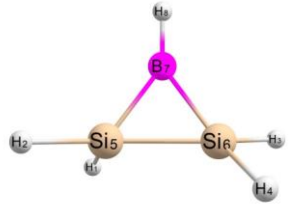

$(3b)^{2-}$(C_2)

(a)

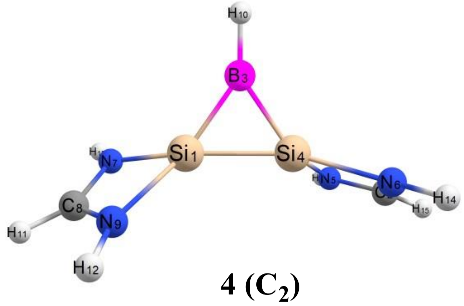

4 (C_2)

(b)

Fig. S16: (a) Structure of model complexes **1-H_2**, **3a**, **3b**, **$3a^{-2}$** and **$3b^{-2}$**; (b) Structure of model compound $(CN_2H_3Si)_2BH$ in *C_2* (**4**).

Table S8. Total energy (hartrees), Relative energy (kcal/mol), ZPE corrected energy (hartrees) and No. of imaginary frequency (NIM) calculated at BP86/6-31G (d,p) level.

Structures	Total Energy (Hartrees)	ZPE corrected Energy (Hartrees)	No of Imaginary Frequency (NIM)
**1** (C_1)	-2579.2650	-2578.2385	0
**1**-H_2 (C_1)	-2580.4828	-2579.4386	0
**2** (C_1)	-3043.3766	-3042.2339	0
**3a** (C_{2v})	-608.0021	-607.9438	2
**3b** (C_{2v})	-606.7569	-606.7162	2
[**3a**]$^{2-}$(C_{2v})	-607.8781	-607.8252	1
[**3b**]$^{2-}$ (C_2)	-606.6445	-606.6071	0
**4** (C_2)	-903.2594	-903.1531	0

Table S9. Comparison of NICS values of $C_3H_3^+$, $Si_3H_3^+$, $(SiH)_2BH$, $(SiH_2)_2BH^{2-}$(**3b**)$^{2-}$, $[Si(NCNH_3)]_2BH$ **4**, structure **1** and **2** at B3LYP/6-311+G (d,p):

Ring Systems	NICS (1)	NICS$_{ZZ}$(0)	NICS$_{ZZ}$(1)
$C_3H_3^+$	-14.7	-30.8	-28.7
$Si_3H_3^+$	-12.7	3.7	-7.1
$(SiH)_2BH$	-13.0	1.9	-9.1
$(SiH_2)_2BH^{2-}$, **(3b)$^{2-}$**	-8.8	7	-8.3
$(CN_2H_3Si)_2BH$, **4**	-13.3	-8.2	-10.5
Si_2B 3-membered ring in **1**	-11.6	-2.0	-8.1
Si_2BN 4-membered ring in **2**	- 4.8	14.1	2.1

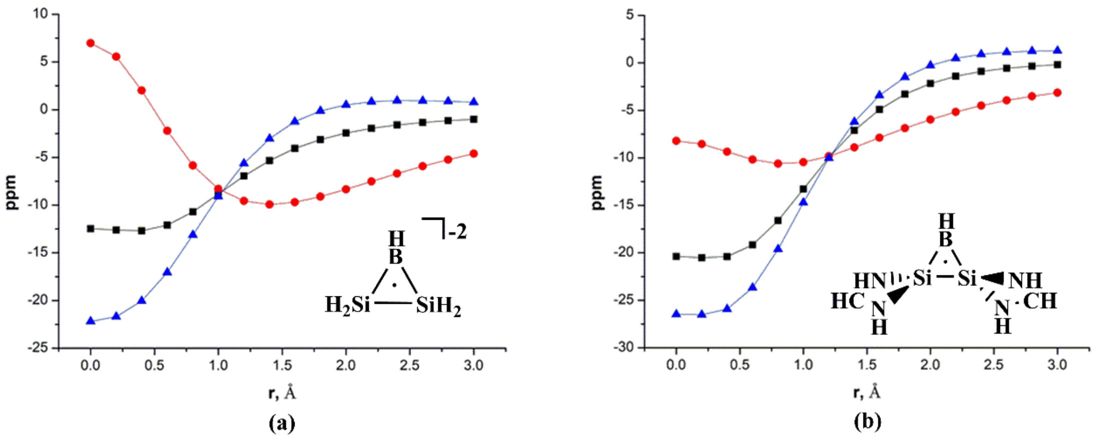

Fig. 17. NICS-scan curves for the model compounds (a) $[(SiH_2)_2BH]^{2-}$, (**3b**)$^{2-}$ and (b) $(CN_2H_3Si)_2BH$, **4**: (black ■) isotropic NICS; (red ●) out-of-plane component; (blue ▲) in-plane component.

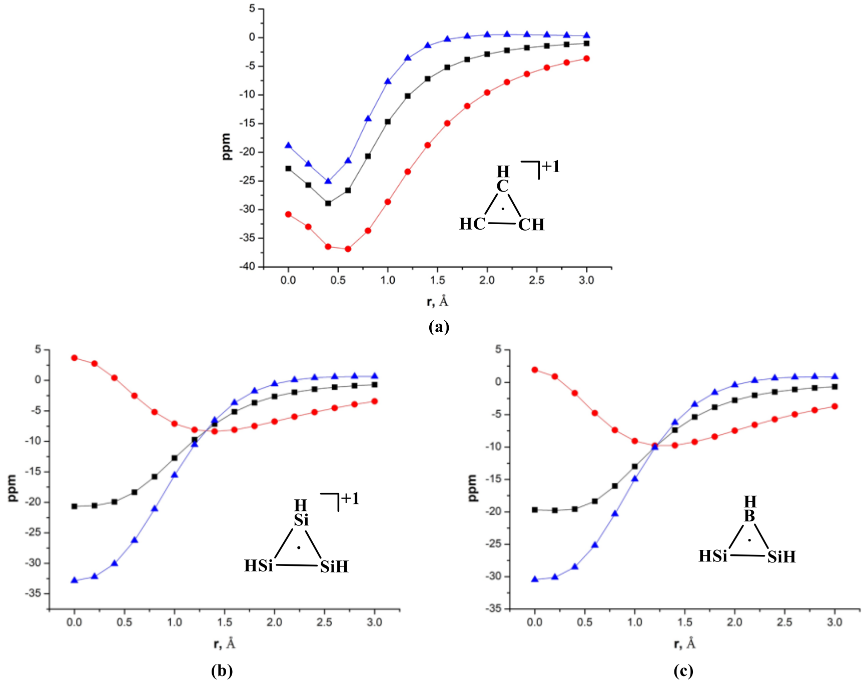

Fig. S18: NICS-scan curves for the three membered cycles (a) $C_3H_3^+$ (b) $Si_3H_3^+$ and (c) Si_2BH_3: (black ■) Isotropic NICS; (red ●) Out-of-plane component; (blue ▲) In-plane component.

Table S10: % Atomic contributions to the π molecular orbitals of structure **2** at BP86/6-31G (d,p)

MO		Si_1	Si_2	B_1	N_1	N_2	N_3	N_4	N_5	OTHERS
HOMO	p	11	10	38	10	-	-	-	-	C_{36}=C_{38}=C_{40}=4
	d	2	2	-	-	-	-	-	-	-
HOMO-6	p	3	3		37	5	5	6	4	C_{19}=9 C_{18}=3 C_{17}=2 Si_3=2
	d	2	2	-	-	-	-	-	-	Si_3=2

*Less than 2% atomic contributions are not considered.

*The numbers in blue signify the labels in the atoms in the crystal structure of **2**

Table S11. Natural Charges on structure **2** based on Natural population Analysis at BP86/6-31G (d,p)

Atom/Fragment	Natural Charge
Si_1	1.722
Si_2	1.729
B_1	-1.026
N_1	-1.726
N_2	-0.716
N_3	-0.699
N_4	-0.689
N_5	-0.716
$Si_1(N_4C_{20}N_5)(C_6H_5)(C(CH_3)_3)_2$	1.289
$Si_2(N_2C_1N_3)(C_6H_5)(C(CH_3)_3)_2$	1.274
$B_1(C_6H_2(C(CH_3)_3)_3)$	-1.389
$N_1Si_3(CH_3)_3$	-1.174

*The numbers in blue signify the labels in the atoms in the crystal structure of

(S5) Cartesian Coordinates.

Structure **1**, (C_1), Singlet, $(LSi)_2BR$[(R = 2,4,6-triisopropylphenyl), (L = $PhC(NtBu)_2$]
Total Electronic Energy: -2579.2650305 au
NIM = 0

Si	-1.032875000000	-0.675622000000	-0.202886000000
C	3.411823000000	-1.222041000000	0.421930000000
B	-0.089071000000	1.009781000000	-0.050447000000
Si	1.146129000000	-0.457346000000	0.227483000000
C	4.809236000000	-1.734995000000	0.555053000000
N	2.621566000000	-1.314518000000	-0.675223000000
C	5.057718000000	-2.960412000000	1.210671000000
H	4.219811000000	-3.525553000000	1.630662000000
N	2.698588000000	-0.588226000000	1.378650000000
C	6.367401000000	-3.450848000000	1.323023000000
H	6.546479000000	-4.405549000000	1.828083000000
N	-2.522781000000	-1.179431000000	-1.333313000000
C	7.443396000000	-2.720521000000	0.793324000000
H	8.464898000000	-3.103082000000	0.884642000000
C	7.203977000000	-1.496749000000	0.146843000000
H	8.038417000000	-0.921271000000	-0.266717000000
C	5.896039000000	-1.006885000000	0.023549000000
H	5.707564000000	-0.059762000000	-0.490931000000
C	2.707224000000	-2.248510000000	-1.830456000000
C	1.717822000000	-1.730195000000	-2.895834000000
H	1.717353000000	-2.410402000000	-3.764577000000
H	2.006010000000	-0.722670000000	-3.238378000000
H	0.697441000000	-1.671156000000	-2.484104000000
C	2.286070000000	-3.666306000000	-1.375145000000
H	2.277290000000	-4.362283000000	-2.232409000000
H	1.275887000000	-3.640739000000	-0.934300000000
H	2.989517000000	-4.062358000000	-0.623077000000
C	4.116147000000	-2.293270000000	-2.462995000000
H	4.858655000000	-2.780541000000	-1.814765000000
H	4.471440000000	-1.277481000000	-2.703185000000
H	4.063445000000	-2.866613000000	-3.404664000000
C	3.148732000000	0.085722000000	2.624800000000
C	1.908249000000	0.775539000000	3.231375000000
H	1.126192000000	0.039614000000	3.479274000000
H	1.475357000000	1.506269000000	2.529541000000
H	2.197523000000	1.300336000000	4.157566000000
C	3.697478000000	-0.937917000000	3.645564000000
H	2.951525000000	-1.727354000000	3.837679000000
H	3.913295000000	-0.429924000000	4.601529000000
H	4.627768000000	-1.411485000000	3.296417000000
C	4.214641000000	1.161606000000	2.312180000000
H	4.471382000000	1.716319000000	3.231350000000
H	3.827230000000	1.883780000000	1.574186000000
H	5.141035000000	0.714190000000	1.918416000000
C	-3.111510000000	-1.867013000000	-0.330182000000
C	-4.383485000000	-2.646194000000	-0.420061000000
C	-5.592964000000	-2.107566000000	0.070746000000
H	-5.593195000000	-1.109847000000	0.519983000000
C	-6.781464000000	-2.846755000000	-0.011142000000
H	-7.713371000000	-2.416699000000	0.369783000000
C	-6.777479000000	-4.133342000000	-0.574828000000
C	-2.240577000000	-2.576148000000	1.986215000000
C	-3.619390000000	-2.868361000000	2.618850000000
H	-3.463187000000	-3.351363000000	3.598939000000
H	-4.181640000000	-1.934763000000	2.786165000000
H	-1.392080000000	-4.523777000000	2.535979000000
H	-2.125198000000	-4.486813000000	0.908389000000
H	-0.539787000000	-3.701316000000	1.191721000000
C	-1.396369000000	-1.791157000000	3.012736000000
H	-1.262952000000	-2.396370000000	3.925598000000
H	-0.406658000000	-1.545105000000	2.595396000000
H	-1.895095000000	-0.846857000000	3.286738000000
C	-0.235270000000	2.577122000000	-0.116875000000
C	0.495960000000	3.354540000000	-1.071281000000
C	0.351771000000	4.754057000000	-1.109751000000
H	0.923890000000	5.332331000000	-1.847941000000
C	-0.504668000000	5.445473000000	-0.238604000000
C	-1.227087000000	4.683036000000	0.696414000000
H	-1.905048000000	5.196006000000	1.391133000000
C	-1.106959000000	3.284406000000	0.774973000000
C	1.452335000000	2.697498000000	-2.070356000000
H	1.340157000000	1.604844000000	-1.941184000000
C	1.102745000000	3.037604000000	-3.535177000000
H	1.767236000000	2.494306000000	-4.231273000000
H	1.217381000000	4.116069000000	-3.745809000000
H	0.061275000000	2.761577000000	-3.771110000000
C	2.922592000000	3.054112000000	-1.760595000000
H	3.193484000000	2.752332000000	-0.734265000000
H	3.100134000000	4.141373000000	-1.845896000000
H	3.609751000000	2.545741000000	-2.461550000000
C	-0.639565000000	6.964625000000	-0.310502000000
H	0.027385000000	7.309968000000	-1.125175000000
C	-0.170615000000	7.646511000000	0.994137000000
H	-0.803549000000	7.347250000000	1.848064000000
H	-0.224555000000	8.746415000000	0.906623000000
H	0.869047000000	7.370014000000	1.236866000000
C	-2.077404000000	7.398861000000	-0.672152000000
H	-2.402908000000	6.946378000000	-1.623759000000
H	-2.145612000000	8.497072000000	-0.770105000000
H	-2.794169000000	7.087503000000	0.108046000000
C	-1.932853000000	2.544590000000	1.831414000000
H	-1.615683000000	1.485808000000	1.793699000000
C	-3.440467000000	2.589570000000	1.500344000000
H	-4.026818000000	2.019742000000	2.244264000000
H	-3.637790000000	2.158432000000	0.503825000000
H	-3.821487000000	3.626757000000	1.495730000000
C	-1.669084000000	3.064810000000	3.260815000000
H	-2.225399000000	2.464552000000	4.003401000000
H	-1.988436000000	4.115494000000	3.382235000000
H	-0.596617000000	3.012165000000	3.513056000000

H	-7.705812000000	-4.710309000000	-0.633697000000
C	-5.578240000000	-4.676085000000	-1.063603000000
H	-5.567442000000	-5.678384000000	-1.503934000000
C	-4.387987000000	-3.936578000000	-0.992801000000
H	-3.454430000000	-4.356664000000	-1.379903000000
C	-3.077785000000	-0.700375000000	-2.626422000000
C	-3.372853000000	-1.884556000000	-3.576117000000
H	-2.471233000000	-2.506224000000	-3.707138000000
H	-3.672624000000	-1.501385000000	-4.566990000000
H	-4.187315000000	-2.522138000000	-3.199179000000
C	-1.998512000000	0.196349000000	-3.269589000000
H	-1.070603000000	-0.368836000000	-3.454156000000
H	-1.750168000000	1.050311000000	-2.619180000000
H	-2.369701000000	0.580597000000	-4.234737000000
C	-4.354916000000	0.141709000000	-2.400334000000
H	-4.698557000000	0.568456000000	-3.358602000000
H	-4.148653000000	0.976286000000	-1.709479000000
H	-5.176043000000	-0.465537000000	-1.987790000000
N	-2.329145000000	-1.726832000000	0.767643000000
H	-4.234424000000	-3.543521000000	2.006500000000
C	-1.529790000000	-3.904473000000	1.632184000000

Structure **1-H$_2$** (C$_1$), Singlet, (LSi)$_2$BH$_2$R[(R = 2,4,6-triisopropylphenyl), (L = PhC(NtBu)$_2$]
Total Electronic Energy: -2580.4828312 au
NIM = 0

Si	1.426376000000	-1.291725000000	0.026995000000
C	-3.873910000000	-1.218178000000	-0.215054000000
B	-0.018693000000	-0.037123000000	0.007186000000
Si	-1.477950000000	-1.259953000000	-0.191378000000
C	-5.367127000000	-1.227603000000	-0.262203000000
N	-3.106560000000	-1.509892000000	0.857969000000
C	-6.061694000000	-2.317430000000	-0.830268000000
H	-5.497363000000	-3.156665000000	-1.248628000000
N	-3.043994000000	-0.929438000000	-1.242133000000
C	-7.463270000000	-2.320013000000	-0.866678000000
H	-7.992002000000	-3.172396000000	-1.305349000000
N	2.981325000000	-1.143603000000	1.133056000000
C	-8.185908000000	-1.231442000000	-0.351346000000
H	-9.279971000000	-1.232669000000	-0.386092000000
C	-7.499762000000	-0.141253000000	0.208794000000
H	-8.057011000000	0.710560000000	0.611752000000
C	-6.098473000000	-0.139467000000	0.261348000000
H	-5.563694000000	0.702317000000	0.711872000000
C	-3.419938000000	-2.392877000000	2.016700000000
C	-2.179383000000	-2.371507000000	2.935356000000
H	-2.343812000000	-3.043347000000	3.794774000000
H	-1.988919000000	-1.356232000000	3.318675000000
H	-1.276219000000	-2.707152000000	2.398793000000
C	-3.662908000000	-3.840796000000	1.526218000000
H	-3.803586000000	-4.517598000000	2.387151000000
H	-2.801479000000	-4.200129000000	0.939527000000
H	-4.567426000000	-3.901118000000	0.898708000000
C	-4.640261000000	-1.894644000000	2.822394000000
H	-5.578923000000	-1.981683000000	2.255562000000
H	-4.507496000000	-0.841659000000	3.120481000000
H	-4.740885000000	-2.500750000000	3.739455000000
C	-3.263605000000	-0.137178000000	-2.483034000000
C	-2.091186000000	-0.488422000000	-3.424607000000
H	1.041639000000	-0.670461000000	2.846221000000
H	2.040417000000	-0.519794000000	4.331529000000
C	3.247056000000	0.987867000000	2.358899000000
H	3.347934000000	1.449369000000	3.356949000000
H	2.330050000000	1.376451000000	1.886198000000
H	4.114375000000	1.302562000000	1.752919000000
N	3.070242000000	-1.422677000000	-1.027099000000
C	3.389307000000	-2.123523000000	-2.302260000000
C	4.667699000000	-1.575277000000	-2.973982000000
H	4.763043000000	-2.013561000000	-3.982630000000
H	4.612607000000	-0.479447000000	-3.079839000000
H	5.578003000000	-1.826020000000	-2.409815000000
C	3.536507000000	-3.643315000000	-2.044474000000
H	3.689177000000	-4.180776000000	-2.996766000000
H	4.403586000000	-3.853193000000	-1.396214000000
H	2.630690000000	-4.044100000000	-1.560910000000
C	2.192871000000	-1.881054000000	-3.246648000000
H	2.344399000000	-2.433277000000	-4.189510000000
H	1.248300000000	-2.221095000000	-2.790656000000
H	2.088756000000	-0.810000000000	-3.483428000000
C	-0.007119000000	1.562926000000	0.122525000000
C	-0.660913000000	2.259263000000	1.196431000000
C	-0.629935000000	3.665381000000	1.276931000000
H	-1.139616000000	4.161173000000	2.114442000000
C	0.028475000000	4.465282000000	0.333328000000
C	0.666664000000	3.795715000000	-0.722775000000
H	1.190141000000	4.385629000000	-1.486769000000
C	0.659013000000	2.393826000000	-0.845208000000
C	-1.425121000000	1.525951000000	2.300945000000
H	-1.315372000000	0.445416000000	2.097166000000
C	-0.845075000000	1.806612000000	3.704804000000
H	-1.370036000000	1.210650000000	4.473855000000
H	-0.950598000000	2.870306000000	3.983935000000
H	0.227366000000	1.556068000000	3.751581000000
C	-2.930714000000	1.867391000000	2.265894000000
H	-3.363640000000	1.640010000000	1.276964000000
H	-3.104332000000	2.939862000000	2.467044000000

H	-2.124335000000	-1.555022000000	-3.705080000000
H	-1.123607000000	-0.289624000000	-2.934064000000
H	-2.154436000000	0.115490000000	-4.345541000000
C	-4.587210000000	-0.482720000000	-3.198337000000
H	-4.671702000000	-1.566779000000	-3.382300000000
H	-4.599789000000	0.029616000000	-4.175706000000
H	-5.472839000000	-0.153077000000	-2.635325000000
C	-3.231185000000	1.367639000000	-2.127967000000
H	-3.324896000000	1.980781000000	-3.041474000000
H	-2.288188000000	1.633073000000	-1.621988000000
H	-4.071195000000	1.626041000000	-1.459897000000
C	3.823134000000	-1.306486000000	0.088102000000
C	5.314455000000	-1.362315000000	0.159398000000
C	6.081920000000	-0.226323000000	-0.177021000000
H	5.575625000000	0.688338000000	-0.500699000000
C	7.481433000000	-0.273701000000	-0.102269000000
H	8.067256000000	0.614637000000	-0.359459000000
C	8.129394000000	-1.455062000000	0.294973000000
H	9.222145000000	-1.490924000000	0.347841000000
C	7.370449000000	-2.590459000000	0.623228000000
H	7.869450000000	-3.514164000000	0.933590000000
C	5.970435000000	-2.545168000000	0.563061000000
H	5.378242000000	-3.424030000000	0.836186000000
C	3.198799000000	-0.553225000000	2.481778000000
C	4.481816000000	-1.069454000000	3.167595000000
H	4.506278000000	-2.171753000000	3.187493000000
H	4.490610000000	-0.710668000000	4.211270000000
H	5.399054000000	-0.707738000000	2.680159000000
C	1.982467000000	-0.981177000000	3.331285000000
H	1.961616000000	-2.077465000000	3.454260000000
H	-3.486351000000	1.291990000000	3.028845000000
C	0.041489000000	5.986600000000	0.454688000000
H	-0.523717000000	6.241563000000	1.373200000000
C	-0.674236000000	6.665683000000	-0.734245000000
H	-0.153513000000	6.452687000000	-1.684515000000
H	-0.701271000000	7.762663000000	-0.606096000000
H	-1.711254000000	6.303903000000	-0.834241000000
C	1.473725000000	6.540756000000	0.622483000000
H	1.977650000000	6.085865000000	1.491653000000
H	1.460250000000	7.636181000000	0.765102000000
H	2.088992000000	6.327839000000	-0.269471000000
C	1.391327000000	1.803975000000	-2.052709000000
H	1.229153000000	0.711353000000	-2.012915000000
C	2.910761000000	2.064778000000	-1.972611000000
H	3.439799000000	1.598847000000	-2.824246000000
H	3.331363000000	1.655067000000	-1.038694000000
H	3.134175000000	3.146768000000	-1.993708000000
C	0.826927000000	2.318614000000	-3.394982000000
H	1.326119000000	1.821967000000	-4.247222000000
H	0.979981000000	3.406409000000	-3.513316000000
H	-0.255572000000	2.124881000000	-3.473237000000
H	1.102203000000	-2.755996000000	0.091124000000
H	-1.180112000000	-2.706735000000	-0.458451000000

Structure **2** (C_1), Singlet, 1-aza-2,3-disila-4-boretidine derivative

Total Electronic Energy: -3043.3766480 au

NIM = 0

Si	1.261149000000	-0.538615000000	0.050847000000
B	-0.030493000000	0.890254000000	-0.005034000000
C	-3.488417000000	-1.281029000000	-0.125101000000
N	0.101012000000	-1.916970000000	0.075558000000
Si	0.233104000000	-3.652947000000	-0.079611000000
C	-5.448108000000	-2.815547000000	-0.512569000000
H	-4.741947000000	-3.641950000000	-0.638581000000
N	-2.633747000000	-1.109613000000	-1.164621000000
N	2.882329000000	-0.976154000000	-0.929615000000
C	-7.732265000000	-1.976510000000	-0.480099000000
H	-8.808036000000	-2.153539000000	-0.576773000000
N	2.745339000000	-0.787000000000	1.237491000000
C	-6.826357000000	-3.040547000000	-0.629192000000
H	-7.193555000000	-4.049708000000	-0.841539000000
C	-1.873791000000	-1.407402000000	3.250678000000
H	-2.091609000000	-1.616449000000	4.311180000000
H	-1.401079000000	-0.414627000000	3.179336000000
H	-1.153009000000	-2.154229000000	2.881567000000
C	-3.176118000000	-1.445364000000	2.427852000000
C	-5.873836000000	-0.455522000000	-0.078391000000
H	-5.500939000000	0.550779000000	0.136040000000
C	-7.252094000000	-0.685894000000	-0.207048000000
H	-7.951449000000	0.148242000000	-0.091294000000
C	-3.863815000000	-2.817787000000	2.610037000000
H	4.119945000000	-0.843357000000	4.403860000000
H	5.107454000000	-0.883056000000	2.923578000000
Si	-1.186472000000	-0.656773000000	0.009612000000
C	-4.959675000000	-1.520260000000	-0.233518000000
N	-2.771601000000	-1.192572000000	1.012847000000
C	1.662559000000	-0.570400000000	3.388685000000
H	1.420690000000	-1.645768000000	3.416097000000
H	0.838316000000	-0.037960000000	2.884071000000
H	1.746770000000	-0.202909000000	4.425074000000
C	3.299958000000	-1.296126000000	-2.324406000000
C	4.404651000000	-0.341848000000	-2.833043000000
H	5.358157000000	-0.489513000000	-2.304295000000
H	4.094257000000	0.708312000000	-2.717678000000
H	4.581325000000	-0.530661000000	-3.906100000000
C	3.794275000000	-2.757412000000	-2.434165000000
H	3.017975000000	-3.465824000000	-2.105152000000
H	4.701583000000	-2.921984000000	-1.830962000000
H	4.043205000000	-2.987394000000	-3.484719000000
C	2.046669000000	-1.110615000000	-3.201471000000
H	2.273744000000	-1.395397000000	-4.242489000000
H	1.710184000000	-0.061999000000	-3.191404000000
H	1.217479000000	-1.737537000000	-2.839559000000
C	-0.087918000000	2.470477000000	-0.068365000000
C	-1.101798000000	3.239976000000	0.610634000000
C	-1.143438000000	4.642684000000	0.527682000000
H	-1.934538000000	5.176160000000	1.071376000000
C	-0.209832000000	5.388973000000	-0.208279000000
C	0.787969000000	4.655958000000	-0.864283000000

H	-3.226353000000	-3.639986000000	2.253937000000
H	-4.829901000000	-2.865267000000	2.084821000000
H	-4.059950000000	-2.979356000000	3.684184000000
C	-4.126704000000	-0.339956000000	2.941805000000
H	-5.093200000000	-0.358900000000	2.414170000000
H	-3.672919000000	0.654031000000	2.818755000000
H	-4.326277000000	-0.497001000000	4.016120000000
C	-2.920457000000	-0.670500000000	-2.563116000000
C	-3.975764000000	-1.559102000000	-3.255280000000
H	-3.706169000000	-2.625739000000	-3.174654000000
H	-4.011446000000	-1.297941000000	-4.326952000000
H	-4.985603000000	-1.420491000000	-2.842478000000
C	-3.371391000000	0.809017000000	-2.563587000000
H	-3.513166000000	1.165249000000	-3.598881000000
H	-2.609174000000	1.442611000000	-2.079861000000
H	-4.329762000000	0.930997000000	-2.031402000000
C	-1.588205000000	-0.793475000000	-3.328909000000
H	-1.255489000000	-1.843732000000	-3.372806000000
H	-0.807949000000	-0.195374000000	-2.827467000000
H	-1.714199000000	-0.424675000000	-4.360690000000
C	1.887985000000	-4.245690000000	0.646288000000
H	1.960676000000	-5.345976000000	0.584782000000
H	1.972999000000	-3.957216000000	1.707471000000
H	2.753528000000	-3.822191000000	0.112175000000
C	-1.135312000000	-4.565051000000	0.869908000000
H	-1.024824000000	-5.655040000000	0.727585000000
H	-2.136323000000	-4.283153000000	0.503304000000
H	-1.095189000000	-4.363161000000	1.953025000000
C	7.066602000000	-2.455629000000	0.727432000000
H	7.507679000000	-3.435660000000	0.935620000000
C	7.889138000000	-1.323888000000	0.600866000000
H	8.974249000000	-1.418559000000	0.708410000000
C	7.313786000000	-0.070363000000	0.336039000000
H	7.948423000000	0.815923000000	0.235722000000
C	0.068612000000	-4.293471000000	-1.869543000000
H	0.142703000000	-5.396083000000	-1.892693000000
H	0.834726000000	-3.895257000000	-2.554312000000
H	-0.918237000000	-4.020481000000	-2.283038000000
C	5.923891000000	0.055086000000	0.194546000000
H	5.476884000000	1.029894000000	-0.023171000000
C	5.093120000000	-1.079132000000	0.325723000000
C	3.608206000000	-0.950949000000	0.209514000000
C	3.296959000000	1.190423000000	2.633003000000
H	3.394042000000	1.561849000000	3.668139000000
H	2.485603000000	1.748990000000	2.137164000000
H	4.245591000000	1.397797000000	2.109876000000
C	2.984148000000	-0.324386000000	2.633739000000
C	5.675891000000	-2.336334000000	0.595657000000
H	5.032745000000	-3.213781000000	0.715709000000
C	4.112706000000	-1.111292000000	3.333317000000
H	3.941402000000	-2.198087000000	3.255593000000
H	1.534740000000	5.208176000000	-1.451587000000
C	0.867773000000	3.252212000000	-0.812166000000
C	2.010127000000	2.619320000000	-1.609258000000
H	1.948001000000	1.527586000000	-1.450312000000
C	1.848964000000	2.875317000000	-3.124816000000
H	2.650263000000	2.382232000000	-3.706076000000
H	1.888368000000	3.954379000000	-3.356944000000
H	0.878256000000	2.494454000000	-3.485218000000
C	3.392005000000	3.099252000000	-1.112963000000
H	3.524965000000	2.881103000000	-0.039236000000
H	3.514229000000	4.189157000000	-1.242750000000
H	4.212690000000	2.613235000000	-1.673017000000
C	-2.165400000000	2.589810000000	1.497919000000
H	-2.023281000000	1.496226000000	1.419766000000
C	-3.598938000000	2.926964000000	1.031803000000
H	-3.756028000000	2.624562000000	-0.017972000000
H	-3.802469000000	4.010642000000	1.093285000000
H	-4.355777000000	2.420756000000	1.658900000000
C	-1.959061000000	2.978844000000	2.979915000000
H	-2.698760000000	2.486554000000	3.638271000000
H	-2.061932000000	4.068904000000	3.124543000000
H	-0.950441000000	2.691551000000	3.321627000000
C	-0.266889000000	6.911132000000	-0.290936000000
H	0.576268000000	7.231458000000	-0.935169000000
C	-0.070779000000	7.573022000000	1.091602000000
H	-0.885109000000	7.293710000000	1.783403000000
H	-0.066030000000	8.674842000000	1.008114000000
H	0.880124000000	7.257141000000	1.552414000000
C	-1.571555000000	7.405682000000	-0.954697000000
H	-1.697005000000	6.968724000000	-1.959536000000
H	-1.575719000000	8.506311000000	-1.052573000000
H	-2.453648000000	7.120010000000	-0.354692000000

Structure **3a** (C_{2v}), Singlet, Si_2BH_7
Total Electronic Energy: -608.0021041 au
NIM = 2

Si	0.000000000000	1.764158000000	-0.133017000000
Si	0.000000000000	-1.764158000000	-0.133017000000
H	0.000000000000	-2.991369000000	0.729379000000
H	-1.205868000000	-1.831842000000	-1.030149000000
H	1.205868000000	-1.831842000000	-1.030149000000
H	1.205868000000	1.831842000000	-1.030149000000
B	0.000000000000	0.000000000000	0.863399000000

H	-1.205868000000	1.831842000000	-1.030149000000
H	0.000000000000	2.991369000000	0.729379000000
H	0.000000000000	0.000000000000	2.069313000000

Structure **3b** (C_{2v}), Singlet, Si_2BH_5
Total Electronic Energy: -606.7569446 au
NIM = 2

H	1.248255000000	1.918514000000	-0.606044000000
H	-1.248255000000	1.918514000000	-0.606044000000
H	1.248255000000	-1.918514000000	-0.606044000000
H	-1.248255000000	-1.918514000000	-0.606044000000
Si	0.000000000000	1.163519000000	-0.258908000000
Si	0.000000000000	-1.163519000000	-0.258908000000
B	0.000000000000	0.000000000000	1.412694000000
H	0.000000000000	0.000000000000	2.610138000000

Structure (**3a)**$^{2-}$ (C_{2v}), Singlet, $(Si_2BH_7)^{2-}$
Total Electronic Energy: -607.8781076 au
NIM = 1

Si	0.000000000000	1.675326000000	-0.115729000000
Si	0.000000000000	-1.675326000000	-0.115729000000
H	0.000088000000	1.581517000000	-1.653740000000
H	-1.135039000000	2.789769000000	0.037622000000
H	1.135039000000	-2.789769000000	0.037622000000
H	-1.135017000000	-2.789780000000	0.037876000000
H	-0.000088000000	-1.581517000000	-1.653740000000
H	1.135017000000	2.789780000000	0.037876000000
B	0.000000000000	0.000000000000	0.860934000000
H	0.000000000000	0.000000000000	2.092221000000

Structure (**3b)**$^{2-}$ (C_2), Singlet, $(Si_2BH_5)^{2-}$
Total Electronic Energy: -606.6444537 au
NIM = 0

H	1.267196000000	1.736565000000	-0.964307000000
H	-0.705036000000	2.663678000000	-0.234386000000
H	0.705036000000	-2.663678000000	-0.234386000000
H	-1.267196000000	-1.736565000000	-0.964307000000
Si	0.000000000000	1.193114000000	-0.248277000000
Si	0.000000000000	-1.193114000000	-0.248277000000
B	0.000000000000	0.000000000000	1.353768000000
H	0.000000000000	0.000000000000	2.580299000000

Structure 4 (C_2), Singlet, $(CN_2H_3Si)_2BH$
Total Electronic Energy: -903.2594425 au
NIM = 0

Si	-0.274357000000	1.071567000000	0.310288000000
C	1.188338000000	-3.073453000000	-0.651493000000
B	0.000000000000	0.000000000000	1.884387000000
Si	0.274357000000	-1.071567000000	0.310288000000
N	1.760203000000	-1.869320000000	-0.607120000000
N	0.000000000000	-2.980702000000	-0.056533000000
N	0.000000000000	2.980702000000	-0.056533000000
C	-1.188338000000	3.073453000000	-0.651493000000
N	-1.760203000000	1.869320000000	-0.607120000000
H	0.000000000000	0.000000000000	3.086334000000
H	-1.621353000000	3.976556000000	-1.105262000000
H	-2.713955000000	1.670888000000	-0.893519000000
H	0.685629000000	3.730913000000	-0.044849000000
H	-0.685629000000	-3.730913000000	-0.044849000000
H	1.621353000000	-3.976556000000	-1.105262000000
H	2.713955000000	-1.670888000000	-0.893519000000

(S6) References

1. S. S. Sen, H. W. Roesky, D. Stern, J. Henn, D. Stalke, *J. Am. Chem. Soc.* **2010**, *132*, 1123-1126.
2. T. Schulz, K. Meindl, D. Leusser, D. Stern, J. Graf, C. Michaelsen, M. Ruf, G. M. Sheldrick, D. Stalke, *J. Appl. Crystallogr*. **2009**, *42*, 885-891.
3. Bruker AXS Inc., SAINT, Madison, **2016**.
4. L. Krause, R. Herbst-Irmer, D. Stalke, *J. Appl. Crystallogr.* **2015**, *48*, 1907-1913.
5. L. Krause, R. Herbst-Irmer, G. M. Sheldrick, D. Stalke, *J. Appl. Crystallogr*. **2015**, *48,* 3-10.
6. G. M. Sheldrick, *Acta Crystallogr.* **2015**, *A71*, 3-8.
7. G. M. Sheldrick, *Acta Crystallogr.* **2015**, *C71,* 3-8.
8. C. B. Hübschle, G. M. Sheldrick, B. Dittrich, *J. Appl. Crystallogr.* **2011**, *44*, 1281-1284.
9. A. Thorn, B. Dittrich, G. M. Sheldrick, *Acta Crystallogr*. **2012**, *A68,* 448-451.
10. M. J. Frisch, G. W. Trucks, H. B. Schlegel, G. E. Scuseria, M. A. Robb, J. R. Cheeseman, G. Scalmani, V. Barone, B. Mennucci, G. A. Petersson, H. Nakatsuji, M. Caricato, X. Li, H. P. Hratchian, A. F. Izmaylov, J. Bloino, G. Zheng, J. L. Sonnenberg, M. Hada, M. Ehara, K. Toyota, R. Fukuda, J. Hasegawa, M. Ishida, T. Nakajima, Y. Honda, O. Kitao, H. Nakai, T. Vreven, J. A. Montgomery, J. E. Peralta, F. Ogliaro, M. Bearpark, J. J. Heyd, E. N. Brothers, K. N. Kudin, V. N. Staroverov, R. Kobayashi, J. Normand, K. Raghavachari, A. P. Rendell, J. C. Burant, S. S. Iyengar, J. Tomasi, M. Cossi, N. Rega, J. M. Millam, M. Klene, J. E. Knox, J. B. Cross, V. Bakken, C. Adamo, J. Jaramillo, R. Gomperts, R. E. Stratmann, O. Yazyev, A. J. Austin, R. Cammi, C. Pomelli, J. W. Ochterski, R. L. Martin, K. Morokuma, V. G. Zakrzewski, G. A. Voth, P. Salvador, J. J. Dannenberg, S. Dapprich, A. D. Daniels, Ö. Farkas, J. B. Foresman, J. V. Ortiz, J. Cioslowski, D. J. Fox, Gaussian 09, Revision D.01. Gaussian, Inc.: Wallingford CT, **2010**.
11. J. P. Perdew, *Phys. Rev. B* **1986**, *33,* 8822-8824.
12. A. D. Becke, *Phys. Rev. A* **1988**, *38*, 3098-3100.
13. F. Weinhold, *J. Comput. Chem.* **2012**, *33*, 2363 -2379.
14. E. D. Glendening, C. R. Landis, F. Weinhold, *J. Comput. Chem.* **2013**, *34*, 1429-1437.
15. K. B. Wiberg, *Tetrahedron.* **1968**, *24*, 1083-1096.
16. T. Lu, F. Chen, *J. Comput. Chem*. **2012**, *33*, 580-592.
17. T. Lu, Q. Chen, *Theor. Chem. Acc*. **2020**, *139*, 25.
18. P. von R. Schleyer, C. Maerker, A. Dransfeld, H. Jiao, N. J. V. E. Hommes, *J. Am. Chem. Soc.* **1996**, *118*, 6317-6318.
19. K. Wolinski, J. F. Hinton, P. Pulay, *J. Am. Chem. Soc.* **1990**, *112*, 8251-8260.
20. (a) A. D. Becke, *J. Chem. Phys.* **1993**, 98, 5648-5652. (b) C. Lee, W. Yang, R. G. Parr, *Phys. ReV. B* **1988**, 37, 785-789.
21. A. Stanger, *J. Org. Chem.* **2006**, 71, 883−893.
